# Adaptation memory in photoreceptors: different mechanisms in rods and cones

**DOI:** 10.3389/fnmol.2023.1135088

**Published:** 2023-04-24

**Authors:** Darya A. Nikolaeva, Maria A. Nekrasova, Alexander Yu. Rotov, Luba A. Astakhova

**Affiliations:** ^1^Laboratory of Evolution of the Sense Organs, I.M. Sechenov Institute of Evolutionary Physiology and Biochemistry RAS, Saint Petersburg, Russia; ^2^Laboratory of Toxinology and Molecular Systematics, L.A. Orbeli Institute of Physiology NAS RA, Yerevan, Armenia

**Keywords:** rod, cone, phototransduction, light adaptation, adaptation memory, dark adaptation

## Abstract

Vertebrate rods and cones operate over a wide range of ambient illumination, which is provided by light adaptation mechanisms regulating the sensitivity and speed of the phototransduction cascade. Three calcium-sensitive feedback loops are well established in both rods and cones: acceleration of the quenching of a light-activated visual pigment and cGMP synthesis by guanylate cyclase, and increased affinity of ion channels for cGMP. Accumulating evidence suggests that the molecular mechanisms of light adaptation are more complex. While investigating these putative mechanisms, we discovered a novel phenomenon, observing that the recovery of light sensitivity in rods after turning off non-saturating adaptive light can take tens of seconds. Moreover, after a formal return of the membrane current to the dark level, cell sensitivity to the stimuli remains decreased for a further 1–2 min. We termed this phenomenon of prolonged photoreceptor desensitization ‘adaptation memory’ (of previous illumination) and the current study is focused on its detailed investigation in rods and an attempt to find the same phenomenon in cones. In rods, we have explored the dependencies of this phenomenon on adapting conditions, specifically, the intensity and duration of adapting illumination. Additionally, we report that fish and frog red-sensitive cones possess similar features of adaptation memory, such as a drop in sensitivity just after the steps of bright light and slow sensitivity recovery. However, we have found that the rate of this process and its nature are not the same as in rods. Our results indicate that the nature of the temporary drop in the sensitivity in rods and cones after adapting steps of light is different. In the rods, adaptation memory could be attributed to the existence of long-lasting modifications of the components of the phototransduction cascade after adapting illumination. In cones, the observed form of the adaptation memory seems to be due to the sensitivity drop caused by a decrease in the availability of the visual pigment, that is, by bleaching.

## 1. Introduction

Retinal photoreceptors, rods and cones can adapt to bright and continuous light and remain partially operable under these conditions. This is possible because of several mechanisms of light adaptation that are activated in the phototransduction cascade under continuous illumination. This two-step cascade of biochemical amplification is based on the regulation of the activity of the effector enzyme cGMP phosphodiesterase (PDE). Photoisomerization of the visual pigment through the activation of G-protein transducin increases the catalytic activity of PDE and decreases the intracellular level of cGMP. cGMP acts as a secondary messenger in this signaling pathway and controls the permeability of ion channels of the outer segment. The mechanisms of light adaptation in photoreceptors regulate the sensitivity to light and the operation speed of the cascade, adjusting the photoreceptor’s operating range to current illumination conditions. Light adaptation is provided by an array of feedback loops that respond to the light-dependent calcium decline in the outer segments occurring after the closure of cGMP-gated channels. There are three generally recognized targets of Ca^2+^ in the phototransduction cascade: (1) low Ca^2+^ concentration accelerates the rate of visual pigment phosphorylation through rhodopsin kinase; (2) a decline in Ca^2+^ level activates the cGMP synthesis by guanylate cyclase (GC); and (3) low Ca^2+^ increases the affinity of the light-sensitive channels for cGMP, as a result, they remain open under lower concentrations of cGMP. These mechanisms counteract the saturation of the photoresponse under intense illumination (Firsov and Govardovskii, 2001; Chen et al., 2010; Arshavsky and Burns, 2012; Vinberg et al., 2018).

Nevertheless, these three main mechanisms of light adaptation are insufficient to explain the entire range of adaptation. In the last few decades, additional putative mechanisms for photoreceptor light adaptation have been proposed. Calvert et al. showed that there is a second quite slow phase of adaptive regulation of sensitivity that accounts for a 40-fold additional decrease (Calvert et al., 2002), and the targets and players of this slow adaptation loop remain unrevealed. In addition, it has been shown that an additional mechanism adjusts the activity of PDE, accelerating the turn-off of its light-induced activity during light adaptation (in the rods of frogs, Astakhova et al., 2008; in the rods of mice, Woodruff et al., 2008). However, the mediators and targets of this PDE activity adjustment remain unknown.

We recently revealed that there is a slow (tens of seconds) process of sensitivity recovery after exposure to background light, up to the pre-exposure level, in frog rods. Interestingly, even after the formal return of the membrane current and, subsequently, the cytoplasmic Ca^2+^ concentration to the dark level, the rod sensitivity to light stimuli remained decreased for a further 1–2 min. This means that the kinetics of this slow process goes beyond the known Ca^2+^-feedback-dependent adaptation mechanisms. We briefly described this phenomenon previously and termed it ‘adaptation memory’, since photoreceptor sensitivity remains decreased as if the cell ‘memorizes’ the previous adapting illumination (Rotov et al., 2021).

Notably, there are several studies describing long-term adaptive changes that persist in mouse rods after steady background illumination. For instance, Krispel et al. revealed that the saturating flashes, delivered as soon as the adapting light had been extinguished and the rods’ circulating dark current had recovered, evoked rod responses with shorter saturation periods than in the dark-adapted state (Krispel et al., 2003). This effect was reversible over time and took tens of seconds for rod responses to recover after turning off the adapting light. That is, this form of adaptation followed a rather slow time course compared to returning cytoplasmic calcium to the dark level. Moreover, the degree of shortening of time in saturation increased with the extension of light exposure time from 10 to 60 s but then the effect reached its limit and did not progress even after 9 min saturating light exposure. It was also shown that the shortening of the response saturation time did not result from a decrease in the gain of the phototransduction cascade and so did not depend on transducin translocation to the inner segment after bright illumination (Sokolov et al., 2002). In another work (McKeown and Kraft, 2014) the authors observed another kind of light-driven effect, a paradoxical form of adaptation in which rods become hypersensitive for some time just after steady background light turn-off, and called this phenomenon an adaptive potentiation. This light-induced increase in photocurrent was demonstrated for both saturated and dim-flash rods’ responses, and the magnitude of potentiation was dependent on the duration of the adapting light exposure. Yet, it should be noted that results obtained by McKeown and Kraft are not in line with our previous data showing long-lasting desensitization instead of potentiation of photoreceptors after light exposure. These findings do not fit into the known Ca^2+^-dependent mechanisms of light and dark adaptation, meaning the mechanisms underlying the adaptation processes in photoreceptors need further research. The present work endeavored to characterize the light adaptation processes in the rods and cones of cold-blooded animals. To gain insight into this matter in more detail we focused on the dependence of the ‘adaptation memory’ effect on the adapting conditions, examining the intensities and lengths of light steps.

## 2. Materials and methods

### 2.1. Experimental animals and preparations

Adult marsh frogs, *Pelophylax ridibundus*, were caught in the wild in southern Russia (Astrakhan District) in September 2022, and Prussian carps, *Carassius gibelio*, were obtained from local hatcheries. The frogs were kept under a water layer in large containers in a refrigerator at 4–6°C. Because the frogs had a greatly reduced metabolic rate at 4–6°C, they did not require food. Two to four days before the experiment the frogs were removed from the refrigerator and exposed to a light: dark cycle of 12 h: 12 h. The fish were kept in an aerated aquarium (40-liter tank containing 5–7 individuals), and the water temperature was maintained at 21–23°C. The fish were fed dry fish food and were also exposed to a 12 h: 12 h light: dark cycle. Prior to the experiment, the animals were dark-adapted overnight. Subsequently, they were euthanized (via decapitation and spinal cord destruction, their eyes were enucleated, and the retinas were extracted under dim red light). All further procedures were conducted under infrared (IR) TV surveillance. During preparations and electrical recordings, the temperature in the experimental room was maintained at 17–19°C.

The handling of experimental animals complied with the requirements of the Directives of the European Community 1986, 86/609/EEC, and the recommendations of the Bioethics Committee of the Institute of Evolutionary Physiology and Biochemistry, Russian Academy of Sciences (Permit-1-13/2022 of January 27, 2022, issued by the Bioethics Committee of the IEPhB RAS).

### 2.2. Solutions

Ringer’s solutions were used for sample preparation, perfusion, and sample storage. The Frog’s Ringer’s solution contained “mM”: NaCl 90, KCl 2.5, MgCl_2_ 1.6, CaCl_2_ 1, NaHCO_3_ 5, HEPES 5, glucose 10, EDTA 0.05, and pH was adjusted to 7.6. The Fish’s Ringer solution contained “mM”: NaCl 102, KCl 2.6, MgCl_2_ 1, glucose 5, CaCl_2_ 1, NaHCO_3_ 28, HEPES 5, 50 mg/l bovine serum albumin (BSA), and the pH was adjusted to 7.8–8.0. A stock solution of sodium aspartate was added to the perfusion solution to a final concentration of 10 mM when required. All the chemicals were purchased from Sigma-Aldrich (St. Louis, MO, United States).

### 2.3. Electrical recordings and experimental protocols

#### 2.3.1. Single-cell recordings

**Figure 1:**
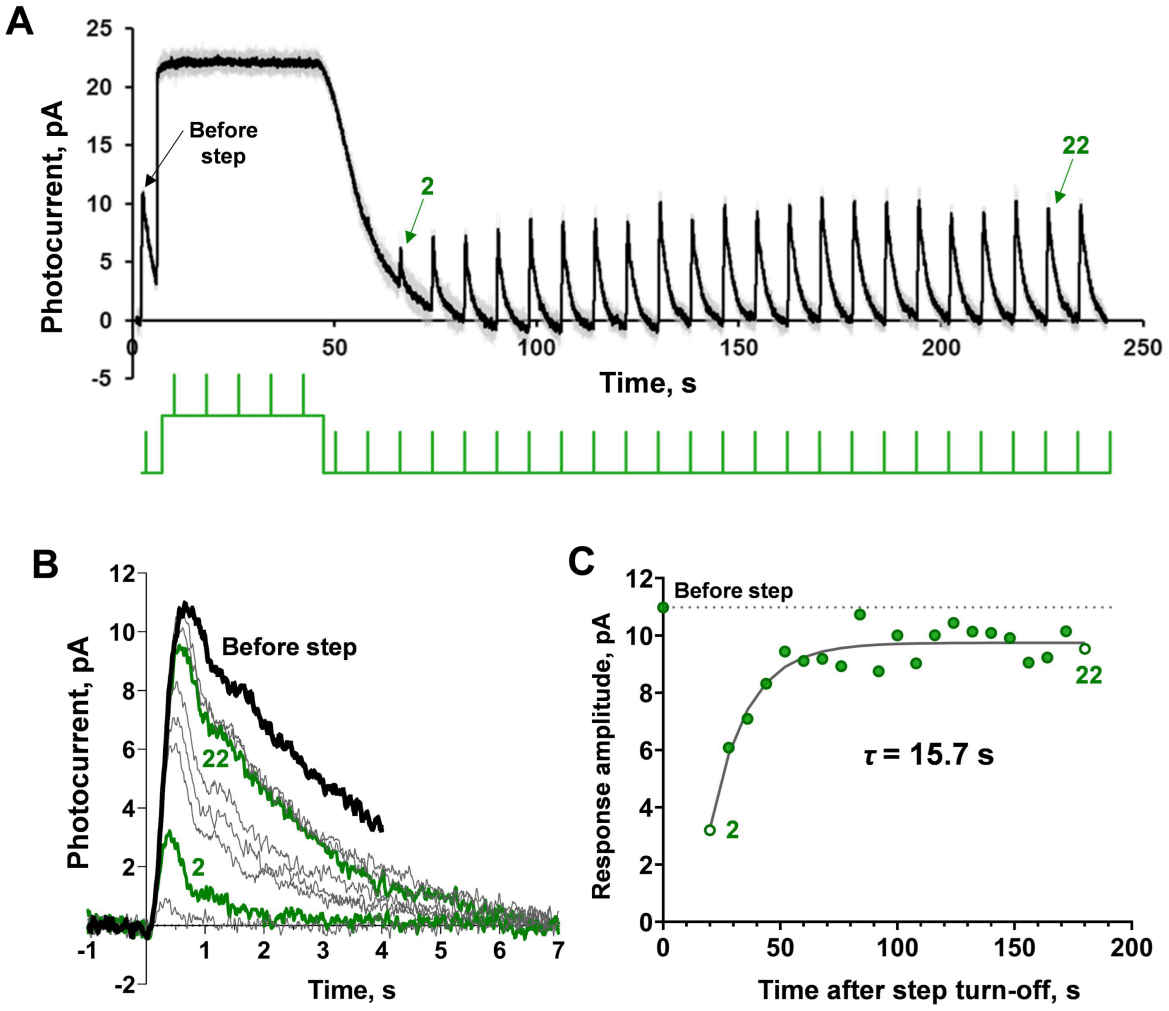
Demonstration of ‘adaptation memory’ phenomenon in frog rods. **(A)** Example of current recording from the isolated frog rod under our light stimulation protocol that combined a saturating light step with brief (2 ms) subsaturating flashes. The black solid line is rod current (averaged from 2 records obtained under the same protocol and smoothed with a moving average filter with a 40 ms window); gray lines represent the original raw recordings; the green line is the light stimulation protocol. **(B)** Single responses cut from record **(A)** and superimposed for shape comparison. The bold black curve corresponds to pre-step photoresponse, and the green bold curves are the second and 22nd photoresponses recorded after the step turn-off. **(C)** Estimation of the amplitude (sensitivity) recovery rate. The dashed line indicates the pre-step level of response amplitude.

The retinas extracted from the eyecups were cut into small pieces using forceps. The resulting suspension of small retinal fragments and isolated photoreceptors was placed in a perfusion chamber of the experimental set-up. Currents of isolated photoreceptors were recorded using a suction pipette (Baylor et al., 1979). Details of the recording set-up and procedures were described previously by Astakhova et al. (2008, 2015). Suction pipette recordings were made from frog rods and fish cones. Rods or cones were sucked into a glass pipette with the outer or inner segments inside, and their currents were recorded under different light stimulus regime. The stimulating light in the suction pipette set-up was provided by a 525 nm wavelength LED. The intensity was regulated stepwise by neutral density filters inserted into the beam and continuously exposed to the LED output, all under computer control. An additional channel used a red (630 nm), green (525 nm), or blue (460 nm) LED (see Supplementary Figure S2A). Light stimulation at two different wavelengths via two independent channels allowed unambiguous identification of the spectral types of fish cones (red-, green-, or blue-sensitive). Data acquisition and light stimulation were conducted using LabView 2016 software and hardware (National Instruments, Austin, TX). Responses were low-pass filtered at 30 Hz and recorded at 2 ms digitization intervals. A typical experimental protocol included the following: (a) recording of responses to stimuli of increasing intensity to determine the working range of the rod or cone; and (b) recording of responses to combinations of prolonged baseline illumination (20–90 s) in one channel and short light stimuli of 2 ms duration delivered at equal intervals in the second channel. An example of such a combined light stimulation protocol is shown in Figure 1A (green line under the rod current record).

#### 2.3.2. Transretinal electroretinogram (ERG) recordings

The isolated retina was mounted in an Ussing-type perfusion chamber with the photoreceptor side up and illuminated with a uniform light field. Responses were recorded using Ag–AgCl electrodes placed on both sides of the retina. The electroretinogram (ERG) *b*-wave was suppressed and the receptor potential was isolated by adding 10 mM sodium aspartate to the perfusion solution. The illuminating system consisted of three independent light channels: one with a 530 nm LED and two with white LEDs. This allowed the application of background light and independently controlled stimulus colors with appropriate filters [see protocol by Wang et al. (2009)]. Specifically, the responses of the frog red-sensitive cones were recorded with orange stimuli (cut-off at 630 nm) using a 530 nm LED steady background that suppressed the rod response (Supplementary Figure S2B). Suppression of the rod component in the ERG-signal was verified by the shape of the response to the short bright flash. For the retina of *C. gibelio*, a 650/660 nm cut-off filter was used to selectively stimulate their red-sensitive cones (Supplementary Figure S2C). The ERG set-up was controlled using the PCI-DAS-1602 I/O card (Measurement Computing, Norton, MA). The controlling program was custom written in the laboratory using Microsoft Visual Basic 96 and Measurement Computing Universal Library. The light stimulation protocol for ERG recordings was similar to that for single-cell recordings: responses to a control semi-saturating flash followed by a continued light exposure (10–90 s) and a subsequent set of flashes to monitor the recovery of the response amplitude.

#### 2.3.3. Light intensity calibration

The emission spectra of all light stimuli used in the setups for single cell and transretinal recordings were recorded using a USB4000 spectrometer (Ocean Optics, United States). The intensities of the LEDs were measured at the same spots where the preparations were located with an OPT-301 optosensor (Burr-Brown Corporation, United States). The light energy measured with an optosensor was then converted into the photon flux density (photons×μm^−2^ × ms^−1^). To compare our stimuli with the absorbance of different photoreceptors, we performed microspectrophotometric recordings from the outer segments of single photoreceptors freely floating in Ringer’s solution between two sealed coverslips. The design of the instrument and procedures for sample preparation and recording have been described previously in detail (Govardovskii et al., 2000; Golobokova and Govardovskii, 2006). After determining the maximum absorbance wavelength for each photoreceptor type, we used the standard nomogram by Govardovskii et al. for further calculations (Govardovskii et al., 2000). To estimate the amount of the bleached pigment produced by an adapting light step, we expressed our stimuli’s intensities in the number of photoisomerizations in a unit cell volume per millisecond of illumination time (R^*^ × μm^−3^ × ms^−1^). In the case of isolated retina recordings, we additionally derived an experimental relation between the decrease in cone sensitivity and the proportion of the pigment bleached. For a detailed description of the bleaching calculation procedure, see Supplementary Material “Calculation of visual pigment bleaching” and Supplementary Figure S1.

### 2.4. Data processing

#### 2.4.1. Formal data analysis

Data captured in the set-up for single-cell recording were processed using custom software written in LabView and then in custom Adaptation Memory software (written in Python by A.A. Zherder, MIKARD-LANA, St. Petersburg). Experimental data captured in the set-up for *ex vivo* electroretinography were processed using custom software written in Microsoft Visual Basic 96 and the Adaptation Memory software written in Python. The Adaptation Memory software reads the long recordings containing the responses of a single cell or a retinal preparation to our protocol combining light steps and a few tens of seconds of brief flashes in a stepwise manner, and smooths the recording by moving the average filter with adjustable window size, to correct the zero line and cut individual responses, and to fit the recovery phases exponentially with the least-square method.

#### 2.4.2. Statistical analysis

Statistical data were processed using Microsoft Excel (Microsoft, United States) and GraphPad Prism 8 (GraphPad Software, United States). The normality of the dataset distributions was confirmed using the Shapiro–Wilk test. The significance of the effects of light exposure step parameters was estimated using two-way ANOVA. Groups of time course parameters for the recovery processes that occurred after the light steps of different intensities or durations were compared using Welch’s ANOVA test with Dunnett’s multiple comparisons test. Additionally, a linear regression analysis was performed to check the significance of the dependencies. Kinetic parameters of responses recorded at the initial and the end phases of the recovery process were compared using a one-way repeated measures ANOVA with Geissner-Greenhouse correction for sphericity, and the Holm-Sidak multiple comparisons test (in the case of more than two groups) or a paired t-test (in the case of two groups). Differences were considered statistically significant at *p* < 0.05. Data in all Figures are presented as individual values with bars depicting the mean ± standard deviation.

## 3. Results

### 3.1. Adaptation memory in frog rods

#### 3.1.1. ‘Adaptation memory’ and dark current recovery kinetics

We have previously described the phenomenon of ‘adaptation memory’ (suppression and slow recovery of light sensitivity long after prolonged exposure to intense light) in isolated rods of marsh frogs (Rotov et al., 2021). In this study, we focused on a detailed estimation of the limits of this phenomenon and the dependence of the rate of current recovery on the duration and intensity of light steps. Furthermore, we analyzed the interconnection between ‘adaptation memory’ and the kinetics of half-saturated responses recorded after the light steps. We used a range of background light intensities from approximately half-saturating (15 photons·μm^−2^·s^−1^) to fully saturating (1,650 photons·μm^−2^·s^−1^) intensity.

The typical results of applying our testing protocol to a single rod are shown in Figure 1A. The first half-saturating test brief flash (2 ms, λ_max_ = 525 nm) was applied before the background light exposure. Then, a 40 s light step (λ_max_ = 525 nm) was turned on, and after its termination, several tens of the same brief test flashes were applied with a total observation period of approximately 200 s (see Figure 1A, where the green line under the current record shows the scheme of light stimulation). In this example, the light step appears to be fully saturating. After the light step is turned off, the rod current quickly returns to the dark level but the amplitudes of the responses to brief flashes continue to recover to the pre-step level over several more tens of seconds. Figure 1B shows the superposition of single responses excised from the record shown in Figure 1A, corrected for the baseline. It can be seen that the amplitude of the response is restored gradually as well as the kinetics of the photoresponse. Complete recovery of light sensitivity was not achieved within 200 s of observation. Figure 1C shows an estimate of the time constant for the rate of adaptation memory after a single-exponential fitting.

**Figure 2:**
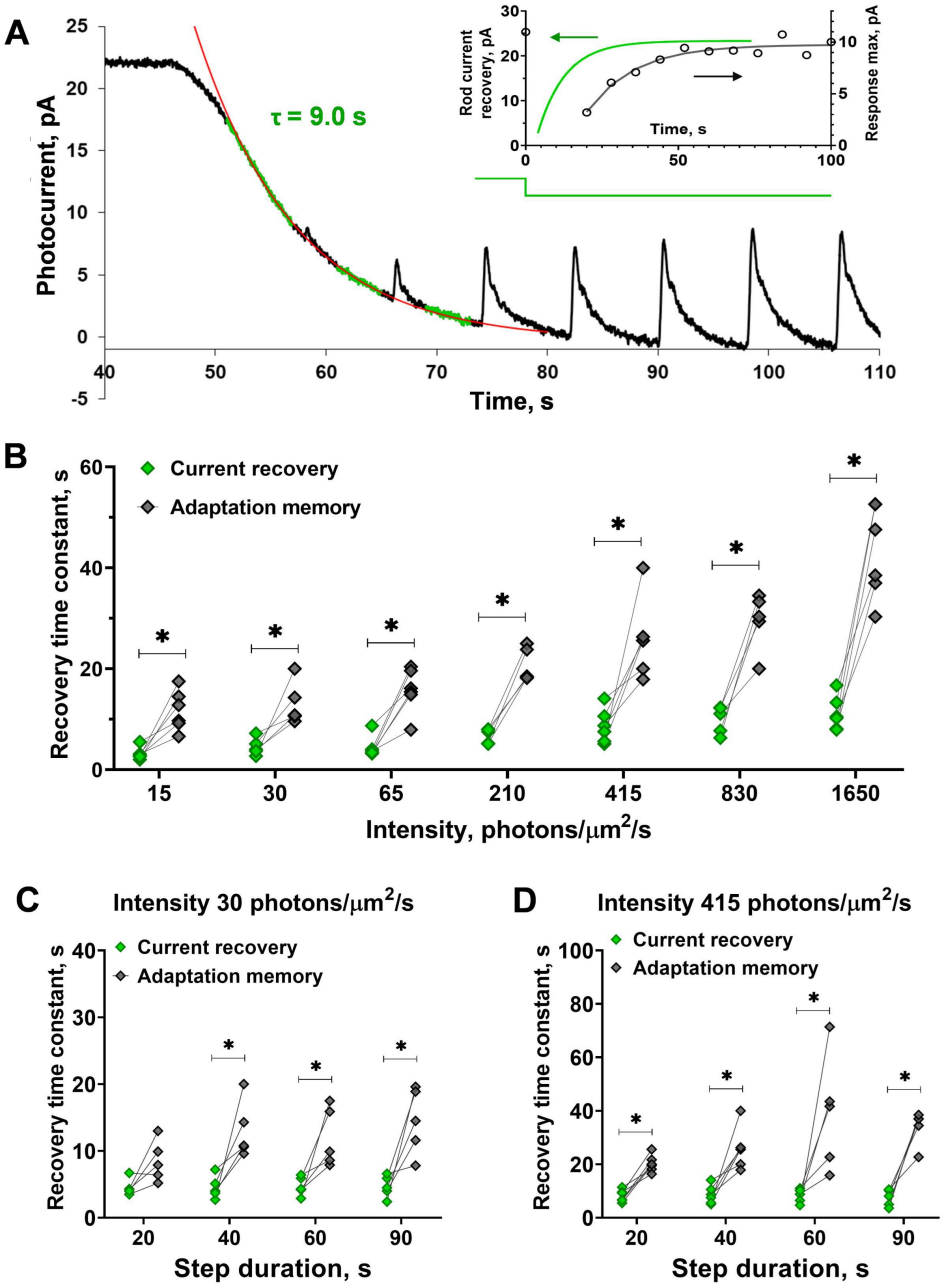
Comparison of the current recovery rate and recovery of sensitivity (‘adaptation memory’) after steps of increasing intensity and duration. **(A)** Evaluation of the current recovery rate with an exponential function. Several fragments between responses to the brief stimuli of the current record were used for approximation (shown by the green noisy line). The red solid line is an exponential fitting for the current recovery phase. Inset shows the time courses of current and rod sensitivity recovery for this cell. The difference in the recovery rates are significant under adaptive illuminations of different intensities **(B)** and durations **(C,D)** except for the one case of a short, low-intensity step. Asterisk ^*^ indicates statistically significant differences between groups (paired *t*-test; *p* < 0.05). Sample sizes *n* = 5–6.

It should be noted that in isolated frog rods, the amplitude of the half-saturated responses shows certain variability. For this reason, we obtained several records (from 2 to 5) for the same protocol for every single rod and then averaged them. We also smoothed the resulting average curves with a moving average filter with a window of 20–30 points. This smoothing procedure minimized noise impact on amplitude maximum estimation without distortion of response shape or position of the maximum. At first glance, the dynamics of subsaturated response recovery after turning off the light step is not the same as the rate of current recovery to its dark level. However, to clearly show that ‘adaptation memory’ differs from the classic dark adaptation, we performed a strict quantitative analysis of the relationship between the rates of these two processes under different parameters of background light steps. To estimate the rate of dark current recovery immediately after the light step, we approximated the declining phase of the response to the step by a single-exponential fitting of several recorded fragments between the responses to the brief flashes, as shown in Figure 2A. In most cases, across the entire range of step intensities and durations, the tested single-exponential fitting was reasonable and provided consistent results for the current recovery rate. A comparison of time constants for rates of flash-response-amplitude recovery and dark-current recovery (Figures 2B–D) demonstrated that the process of ‘adaptation memory’ is not the same. This process is not the same as the mechanisms underlying dark current recovery, and therefore, cannot be accounted for by the classical, well-established loops of light adaptation in rods. In general, both the time constants increase with an increase in the intensity of applied light steps and differ from one another significantly within the entire range of these parameters (two-way ANOVA, *p* < 0.0001 for light intensity as the source of variation; Figure 2B and Supplementary Figure S3). This was as high as several fold in the upper part of the tested range of step intensities when they started to fully saturate the frog rods.

Analysis of the step duration effect on different magnitudes between the rates of current and the response amplitude recovery revealed no impact (Figures 2C,D; two-way ANOVA; *p* = 0.19 and 0.07 for light intensities of 30 and 415 photons·μm^−2^·s^−1^, respectively). However, interestingly, we found that for one particular step (20 s of 30 photons·μm^−2^·s^−1^) the time course of ‘adaptation memory’ did not differ from dark current recovery (paired *t*-test; *p* = 0.07). This result suggests that the ‘adaptation memory’ mechanism in rods does not develop or could be very weak under short low-intensity light steps and requires a certain intensity level and duration of adaptive light.

#### 3.1.2. Dependence of ‘adaptation memory’ course on illumination parameters

**Figure 3:**
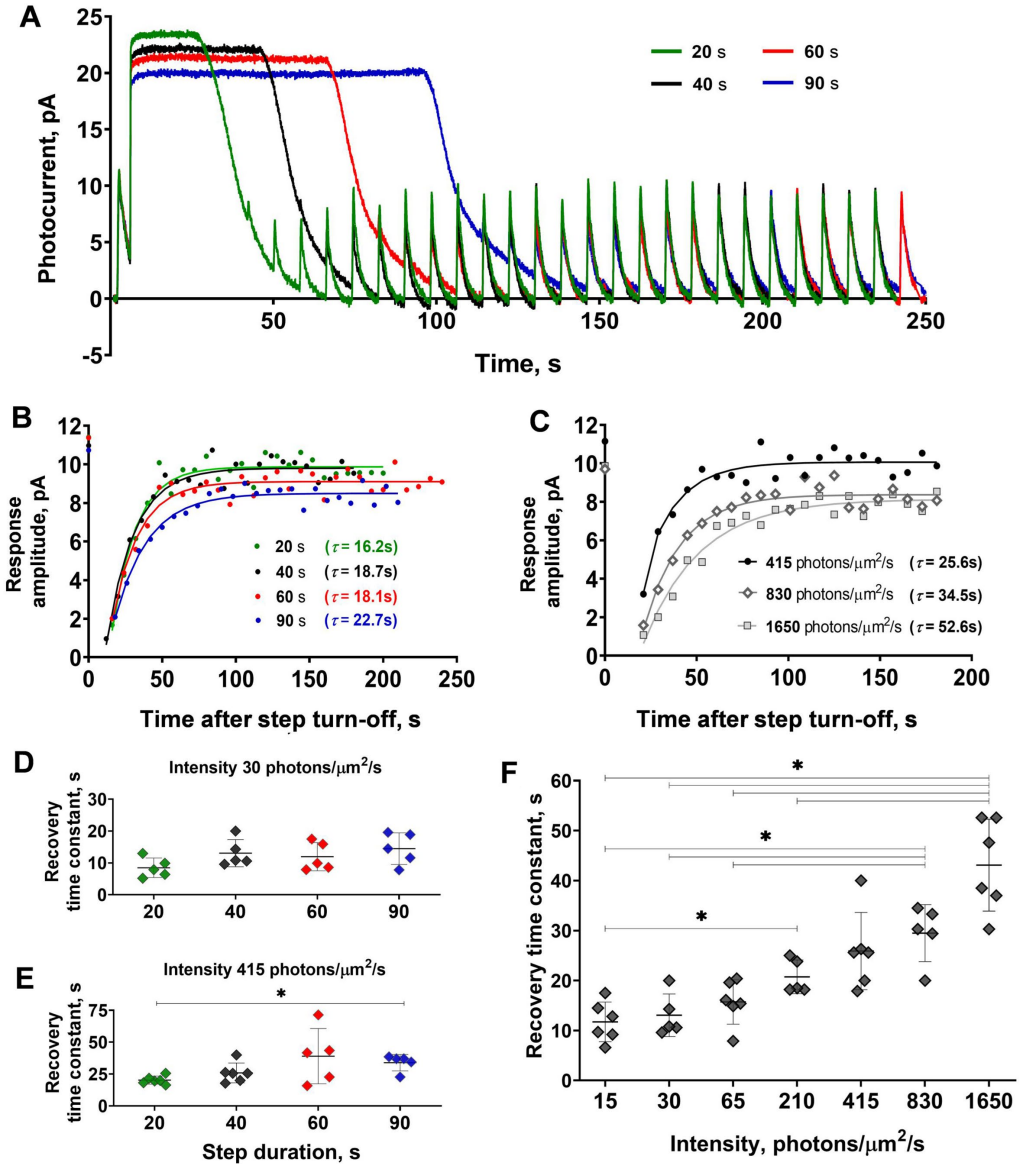
Comparison of the sensitivity recovery rate after light steps of increasing duration and intensities. **(A)** Responses of the representative isolated frog rod to stimulation with a combination of saturating light exposure steps of different durations and the same 2 ms light stimuli (every curve averaged from 2–5 records obtained under the same protocol conditions and smoothed with moving average filter with a 40 ms window). Black line –40 s step; green –20 s step; red –60 s; and blue –90 s step. **(B)** Dynamics of response amplitudes after steps from panel A. **(C)** Dynamics of response amplitudes after 40 s steps of four different intensities (210, 415, 830, and 1,650 photons·μm^−2^·s^−1^). In panels **(B)** and **(C)** for reference, the point plotted at time zero illustrates the pre-step value. Changes in step duration do not affect the time course of current recovery for both moderate intensities **(D)**, while saturating intensity steps **(E)** show a significant difference between the 20 and 90 s illumination lengths. An increase in 40 s stimulus intensity leads to a significant slowdown in the current recovery **(F)**. Asterisk ^*^ indicates statistically significant differences between groups (Dunnett’s multiple comparisons tests; *p* < 0.05). Sample sizes *n* = 5–6.

For the next step of our study, we analyzed the dependence of the rate of ‘adaptation memory’ on the duration and intensity of the adaptive light steps. The results from one isolated frog rod are shown in Figures 3A–C. We used light steps of four different durations, 20, 40, 60, and 90 s, and compared the time constants of response amplitude recovery. The responses of the representative isolated frog rod to a combination of saturating light steps of these lengths and brief 2 ms test flashes are shown in Figure 3A. The time constants obtained for this rod are provided in Figure 3B, and a slight increasing trend in the recovery time constant was observed with increasing step duration (two-way ANOVA; *p* < 0.05 for the duration as the source of variation). It should be noted that a minor loss of current over time is observed in this case. Single rods of marsh frogs are quite stable in long-lasting recording protocols but they can show some decline in dark current with time. We considered that dark current variation up to 20% is normal for considering the rod’s physiology as unaltered and we suppose that is not the result of not waiting long enough between steps. The results of statistical analysis for all tested rods are shown in Figures 3D,E, and the difference in recovery time constants was significant between the two outermost step durations (20 and 90 s; Dunnett’s test, p < 0.05). It should be noted that this difference is observed only under high (415 photons·μm^−2^·s^−1^), but not low (30 photons·μm^−2^·s^−1^) intensity of the adaptive step, moreover, the linear regression slope was insignificant in both cases. The results of the comparison of the ‘adaptation memory’ rates under different intensities of light steps are shown in Figures 3C,F. We found that the intensity of the adaptive background light significantly affected the time constant of the sensitivity recovery course (two-way ANOVA; *p* < 0.0001 for intensity as the source of variation). This dependence is positively correlated according to the linear regression test (slope coefficient = 0.02 photons^−1^·μm^2^·s^2^, *p* < 0.001, R^2^ = 0.95), therefore, the highest intensity of the adaptive step led to the slowest recovery of the response amplitude.

**Figure 4:**
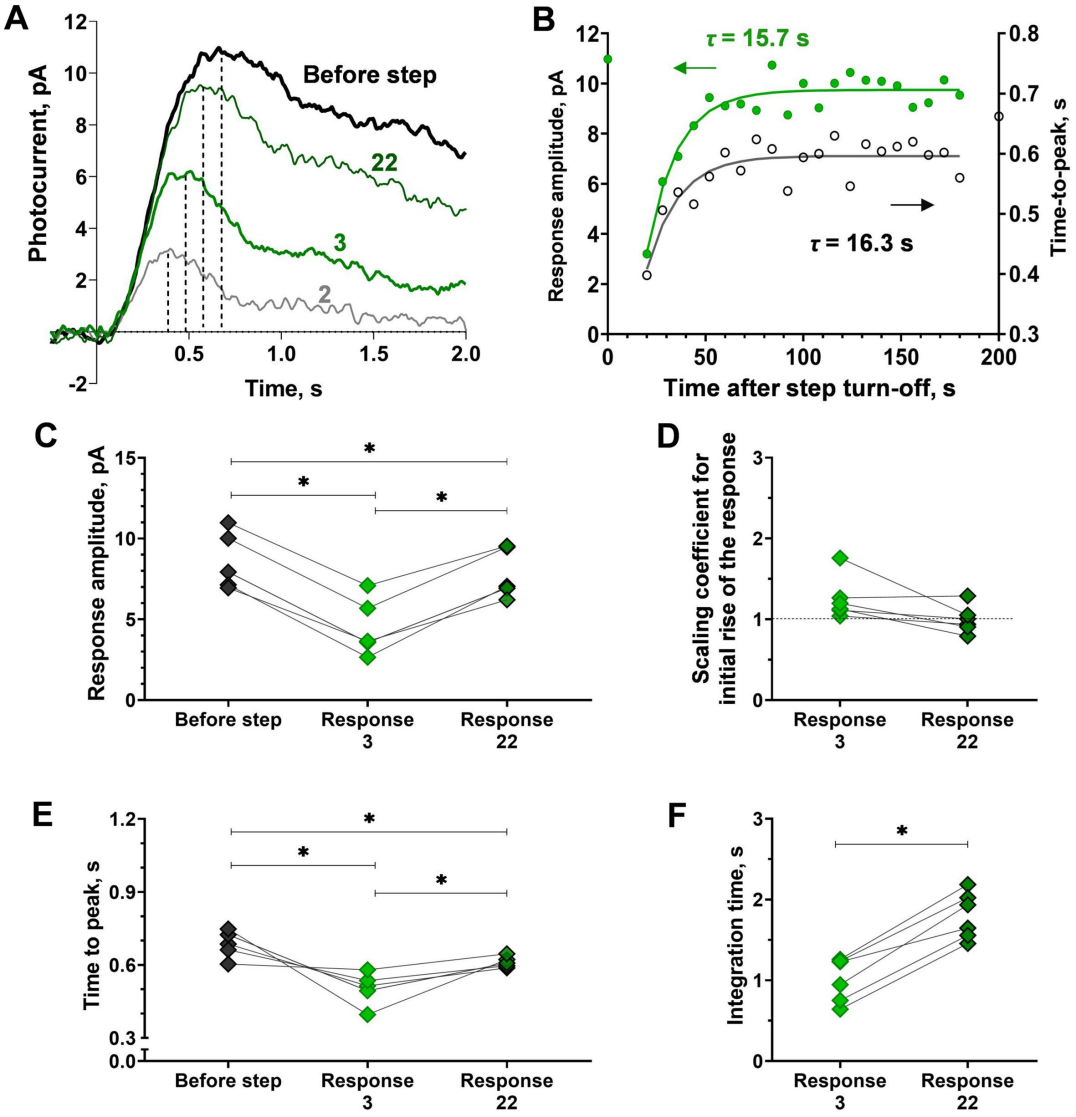
Analysis of response kinetics in relation to ‘adaptation memory’ in isolated frog rods. **(A)** Superimposing of selected responses to the same brief test flashes recorded at different time points after the step turn-off cut from the recording presented in Figure 1A. The bold black curve corresponds to pre-step photoresponse, green curves are the 3rd and 22nd photoresponses recorded after the step turn-off. Dashed lines illustrate the time-to-peak values. **(B)** Difference in the rate of sensitivity recovery (green circles, left Y-scale) and the time-to-peak recovery rate black empty circles, right Y-axis). For reference, the point plotted at time zero illustrates the pre-step value. **(C-F)** Difference in response amplitude **(C)** to several response kinetic parameters: scaling coefficient for rising phase **(D)**, time-to-peak **(E)**, and integration time **(F)** for pre-step response (‘before step’), early response after the step turn-off (‘Response 3’) and last observable response the step turn-off (‘Response 22’). Asterisk * indicates statistically significant differences between groups (Holm-Sidak or paired t-test, *p* < 0.05). Sample sizes *n* = 6.

Visual inspection of superimposed sets of responses to the same brief test flashes recorded at different time points after turning off the 40 s of 415 photons·μm^−2^·s^−1^ step suggests that suppression and the subsequent slow recovery of response amplitude strongly correlate with changes in response kinetics. At first glance, there were no significant changes in the initial phase of the photoresponse, whereas the flash response time-to-peak and kinetics of the response to the turning off the step changed markedly. We estimated this point in more detail and the results of our analysis are shown in Figure 4. First, we analyzed how time-to-peak changed after step turning-off during the post-step observation period, and in general, the time course of time-to-peak recovery was similar to that of amplitude recovery (Figure 4B). This suggests that the adaptive step led mainly to accelerating the response turn-off, and this factor is the main contributor to amplitude suppression and the subsequent slow recovery. To test this, we performed a systematic analysis of the initial phase slopes, time-to-peak, integration times, and amplitudes of photoresponses to the same brief test flashes recorded at two different time points after step turning-off, for response #3 (35 s after step turn-off, the first point when dark current, in most cases, is fully recovered) and #22 (531 s after step turning-off). To estimate the possible relationship between ‘adaptation memory’ and the activation rate of the phototransduction cascade, we analyzed the behavior of the initial phase of the response. For the purposes of the present study, we only needed to determine the relative changes in the steepness of the initial phase. Therefore, we made no assumption regarding the mathematical descriptions of the biochemical mechanisms underlying the rising phase and extracted the ratio of the two activation rate parameters by adjusting the rising phases of these two photoresponses. Figure 4D shows the scaling coefficients that indicate no change in the rising phase steepness for responses #3 and #22 in relation to the rising phase of the pre-step response (paired *t*-test, *p* = 0.06). These scaling coefficients do not significantly differ from 1 (one-sample *t*-test; *p* = 0.07 and 0.97 for responses #3 and #22, respectively), indicating that there is no involvement of activation processes in the ‘adaptation memory’ mechanism. Simultaneously, we revealed significant changes in the amplitudes and time-to-peak of responses #3 and #22 in comparison with the pre-step response (repeated measures ANOVA; *p* < 0.01), additionally, there is а marked and a statistically significant decrease in the integration time of the responses after the light step (paired *t*-test; *p* < 0.01; Figures 4C,E,F). Together, the ‘adaptation memory’ seems to affect the quenching phase of the phototransduction cascade.

### 3.2. Search for adaptation memory in isolated fish cones

As previously mentioned, we were interested in whether a similar phenomenon of ‘adaptation memory’ (prolonged desensitization) occurs in another type of vertebrate photoreceptor, cones. As we have had experience in working with single cones of Prussian carp, *Carassius gibelio* (Astakhova et al., 2015; Rotov et al., 2017) this object was chosen to search for potential ‘adaptation memory’ in single cones.

The retina of *Carassius gibelio* possesses four spectral types of cones: red-, green-, blue-, and UV-sensitive (Govardovskii et al., 2000). The combination of stimulating light sources in our set-up allowed us to identify three of them (red-, green-, and blue-sensitive; Supplementary Figure S2B), and we applied the stimulation protocol for ‘adaptation memory’ testing in all three types. It should be noted that cones are several orders of magnitude less sensitive than rods, and previously we showed that the difference in sensitivity is about 2.5 orders of magnitude between frog rods and green-sensitive *C. gibelio* cones and approximately three orders of magnitude between rods and red-sensitive cones (Astakhova et al., 2015; Figure 2). Considering this, for single cones, we used adaptive light steps of higher intensities by approximately two orders of magnitude.

**Figure 5:**
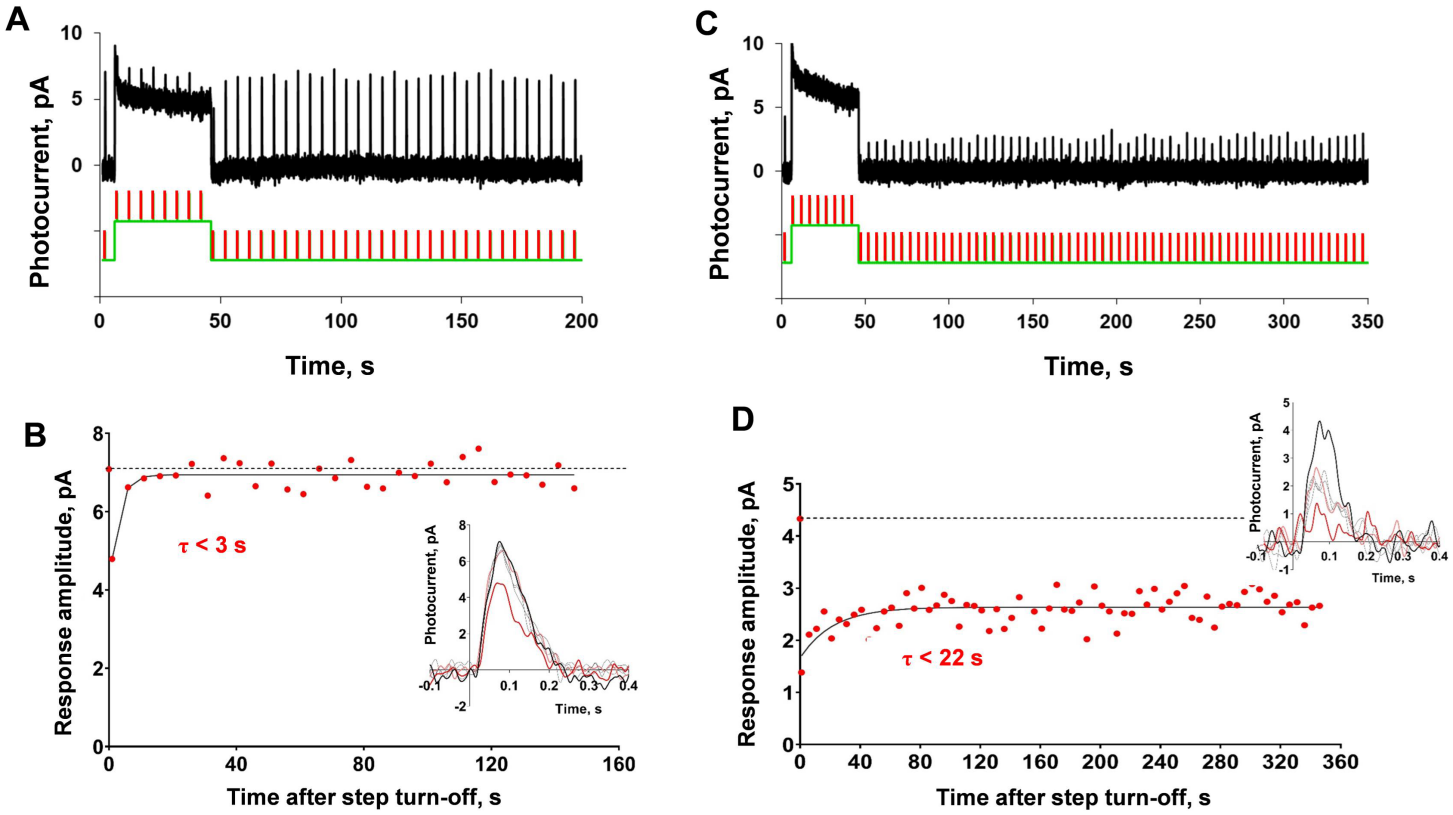
Analysis of ‘adaptation memory’ in a single red-sensitive cone of *Carassius gibelio*. **(A)** Response representative of the cone compared to the combination of the green light step (duration 40 s, intensity 6.5×10^4^ photons·μm^−2^·s^−1^, 525 nm) with brief light flashes (2 ms, intensity 8,170 photons·μm^−2^, 630 nm), and **(B)** shows dynamics of the response amplitude recovery after the initial step. **(C)** Recordings from the same cone under the same protocol of stimulation but the intensity of the light step is one order of magnitude higher (duration 40 s, intensity 8.2×10^5^ photons·μm^−2^·s^−1^, 525 nm) and **(D)** dynamics of the response amplitude recovery for the record **(C)**. In panels **(B)** and **(D)** for reference, the points plotted at time zero and dashed lines illustrate the pre-step value.

The typical results of this experiment are shown in Figure 5. Generally, the first testing light steps for certain cones were less intensive, and in most cases, the amplitude of response was lowered only for the first response after the initial step and then recovered extremely quickly, providing a time constant for amplitude recovery of 1–2 s (Figures 5A,B). We interpreted this as the absence of significant ‘adaptation memory’ and gradually increased the light step intensity based on the result for a single rod, which suggests the appearance of ‘adaptation memory’ only at certain intensities of the adaptive step and its absence at low intensity. The main effect of this increase in adaptive step intensity was the irreversible drop in sensitivity after step turning-off, as shown in Figures 5C,D. This example demonstrates the behavior of a *C. gibelio* red-sensitive single cone; however, the same pattern was observed for cones of all three spectral types in several tested animals (data not shown). The variability in response amplitude in this state becomes close to the signal-to-noise ratio, which makes any further estimations of the possible effects of ‘adaptation memory’ pointless.

We concluded that this finding could be attributed to the bleaching of a significant amount of visual pigment during the high-intensity light step that a single cone cannot regenerate. For the cone visual cycle, Müller glial cells are extremely important, because all-trans-retinol from cones is isomerized to 11-cis-retinol specifically in Müller cells, returns to cones, and is transformed into cis-retinal and binds to opsin, which leads to regeneration of the visual pigment (Wolf, 2004; Kaylor et al., 2013). It should be noted that, in the case of fish cones, we recorded currents from either isolated pairs (for red-or green-sensitive cones) or single isolated cones (for blue-sensitive cones), therefore, there were no fragments of the retinal tissue surrounding them. When applying the suction pipette technique to isolated cones, which are separated from the rest of the retinal tissue, including Müller cells, regeneration is impossible, and bleaching is irreversible. Moreover, the post-bleach sensitivity recovery is blocked even for cones attached to a small piece of the retina if their outer segments are drawn into a suction pipette (Wang et al., 2009). Thus, we cannot trace how a cone reacts in terms of sensitivity to bright background illumination because of the strong limitations for sensitivity that are not relevant for photoreceptors in a living eye. Based on this, we moved on to the recordings of cone responses from the entire isolated retina to observe the effect of background illumination on sensitivity under conditions where bleaching is not irreversible.

### 3.3. Drop and slow recovery of sensitivity in red-sensitive fish cones within the isolated retina

**Figure 6:**
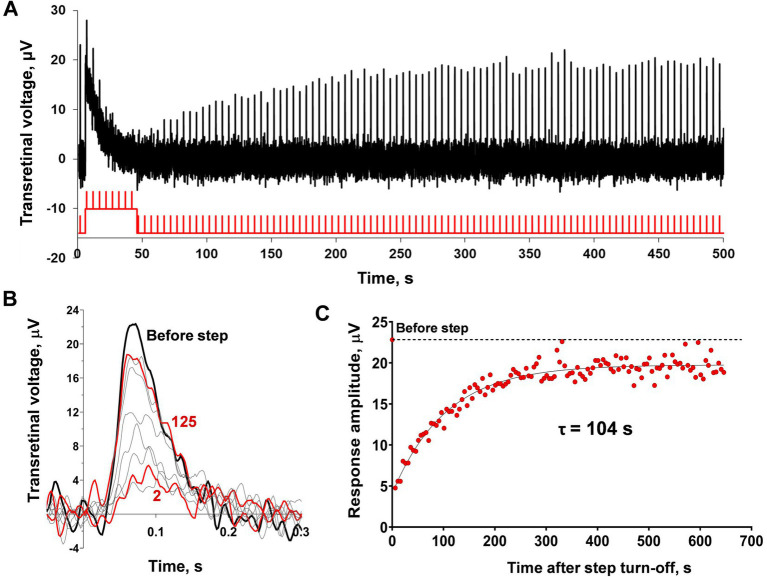
Analysis of sensitivity recovery after the light step in *C. gibelio* red-sensitive cones. **(A)** Example representative of the ERG-recordings from the isolated *C. gibelio* retina after application of 10 mM sodium aspartate and the continuous green background light (33,600 photons·μm^−2^·s^−1^). The testing step and brief flashes were done with red light (see Material and Methods) with intensities of 3.4×10^5^ photons·μm^−2^ for flashes and 1.08×10^7^ photons·μm^−2^·s^−1^ for step. **(B)** Single responses cut from the recordings **(A)** and superimposed for shape comparison: black bold line indicates the pre-step response, red bold lines indicate the first response after the step and the final response after the step during the observation period (626 s after the step turn-off), thin gray lines indicate several intermediate responses. **(C)** Evaluation of the rate of response amplitude recovery with an exponential function. For reference, the point plotted at time zero and the dashed line illustrates the pre-step value.

To eliminate the irreversible bleaching in cones under bright light steps that are expected to cause the ‘adaptation memory’ in cones, we performed the experiments on the isolated *C. gibelio* retinas. Using sodium aspartate, we extracted the photoreceptor component of the ERG, and then the continuous green background light was applied to saturate rods in the retina. The Müller cells-derived ERG component was also suppressed by the green background since we observed responses with fast kinetics typical for cones (Figure 6). Combining these conditions with red light stimuli allows the extraction of only the red-sensitive cone component.

This approach provides cones with the ability to function under bright light steps in conditions close to the living eye state. The typical result of applying the combination of a 40 s light step and testing with the same brief flashes to the isolated *C. gibelio* retina is shown in Figure 6. The light step led to a marked drop and a subsequent slow recovery of the response amplitude. The ability of cones within the retinal tissue to recover sensitivity after fractional bleaching has already been reported for different vertebrate species (amphibians –Goldstein, 1970; Hood and Hock, 1973; Wang et al., 2009; mammals –Wang and Kefalov, 2009). This effect resembles ‘adaptation memory’ in single frog rods apart from the fact that the rate of sensitivity recovery is much slower. The time constant, in this case, was approximately a hundred seconds (Figure 6B). Another difference is that the response kinetics does not demonstrate a marked shift of the time-to-peak although the analysis is hampered by noisy responses.

Generally, the *C. gibelio* retinal preparations were not a good object for the detailed study of the effect of ‘adaptation memory’ on red-sensitive cones because most preparations showed very low amplitudes of responses and an unsatisfactory signal-to-noise ratio, preventing the extraction of any consistent data. Therefore, we decided to perform a similar electroretinographic series on the retinas of the marsh frogs.

### 3.4. Drop and recovery of sensitivity for the extracted response of red-sensitive frog cones from the isolated retina

**Figure 7:**
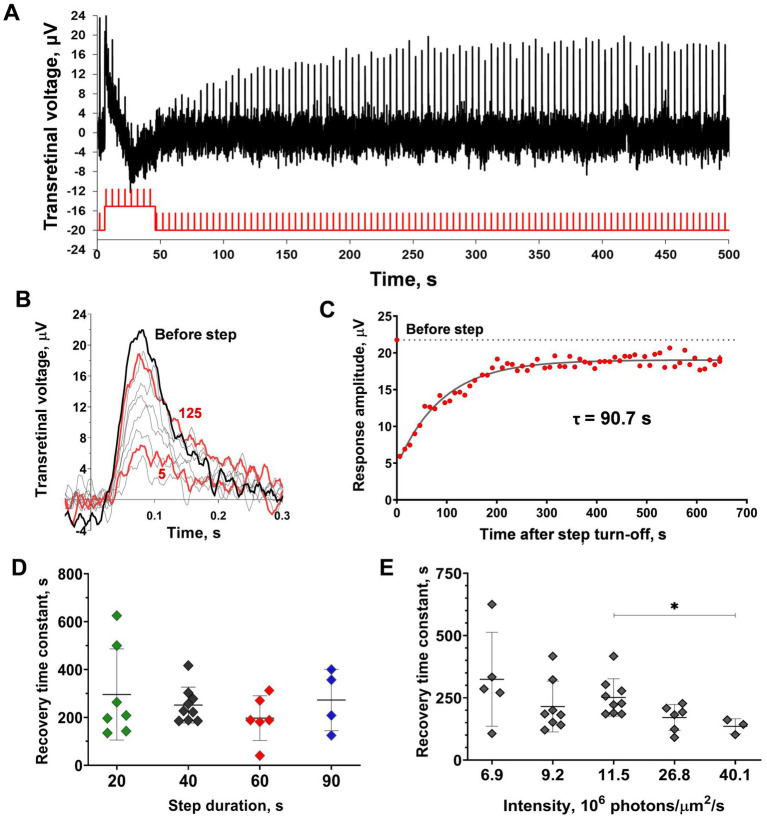
Analysis of sensitivity recovery after the light step in marsh frog red-sensitive cones based on *ex vivo* ERG. **(A)** The representative ERG-recording from the isolated frog retina after application of 10 mM sodium aspartate and the continuous green light (43,400 photons·μm^−2^·s^−1^). Testing step and brief flashes were performed with red light (see Material and Methods) with intensities of 8×10^5^ photons·μm^−2^ for flashes and 26.8×10^7^ photons·μm^−2^·s^−1^ for step). **(B)** Single responses cut from recordings **(A)** and superimposed for shape comparison: black bold line indicates the pre-step response, red bold lines indicate the 5th and 125th responses after the initial step during the observation period (626 s after the step turn-off), thin gray lines indicate several intermediate responses. **(C)** Evaluation of the rate of response amplitude recovery with an exponential function. For reference, the point plotted at time zero and the dashed line illustrates the pre-step value. **(D,E)** Comparison of the sensitivity recovery rate after light exposure steps of different duration (D; 20, 40, 60, and 90 s) and intensities **(E)**. Asterisk ^*^ indicates statistically significant differences between groups (Dunnett’s multiple comparisons test; *p* < 0.05). Sample sizes *n* = 5–9.

We used a similar experimental protocol for the isolated frog retinas (See Supplementary Figure S4) and they demonstrated similar behavior (Figures 7A–C) but with better, long-lasting performance in the experiments and a better signal-to-noise ratio. These satisfactory characteristics allowed us to perform a consistent evaluation of the dependence of the time course of cone ‘adaptation memory’ on the intensity and duration of the adaptive light step and the involvement of response kinetics in this process. It should be noted that the frog retina contains two types of red-sensitive cones, single and double ones (Röhlich and Szél, 2000). However, there is no evidence that their response sensitivity and kinetics differ in any way and the cone response extracted from the ERG is similar to the response of isolated single cones (Rotov et al., 2017).

Figure 7D shows how the duration of the adaptive light step affects the rate of post-step response recovery. No significant differences were found by this factor; furthermore, there were no significant trends (two-way ANOVA; *p* = 0.48 duration as the source of variation). The dependence of cone ‘adaptation memory’ on adaptive step intensity is shown in Figure 7E. It should be noted that the intensity range of the tested light steps was not as broad as that for single rods because of limitations in our experimental setup for *ex vivo* ERG: in the lower limit of the range due to the absence of the ‘adaptation memory’ effect, and upper limit of the range due to the capabilities of our experimental light sources. In this relatively narrow intensity range of steps, a slight dependence on the response recovery rate was observed, and there were significant differences between the intensities of 11.5 and 40.1 photons·μm^−2^·s^−1^ (Dunnett’s test; *p* < 0.05). Surprisingly, this dependence is opposed to that revealed in single rods; the increase in intensity accelerates the recovery rate. The linear regression slope was close to significance though not reaching this level (slope coefficient = −4.5 × 10^−6^ photons^−1^ × μm^2^ × s^2^, *p* = 0.055, R^2^ = 0.76).

We attempted to estimate the amount of bleached pigment in the frog cones despite the fact they cannot provide robust responses isolated from the retina (unlike the tiger salamander cones studied by Kefalov et al., 2005), meaning any calibration experiments were restricted to the ERG recordings and, therefore, were limited to provide an accurate estimation of bleaching. However, we managed to calculate a certain value of pigment bleaching with adaptive light steps in relation to the cone state under the continuous rod-suppressing background (for details, see Supplementary Material). It appeared that in our experiments the bleaching varied between 6 and 34%, while the rod-suppressing background dropped the pigment amount by approximately 1.7%.

**Figure 8:**
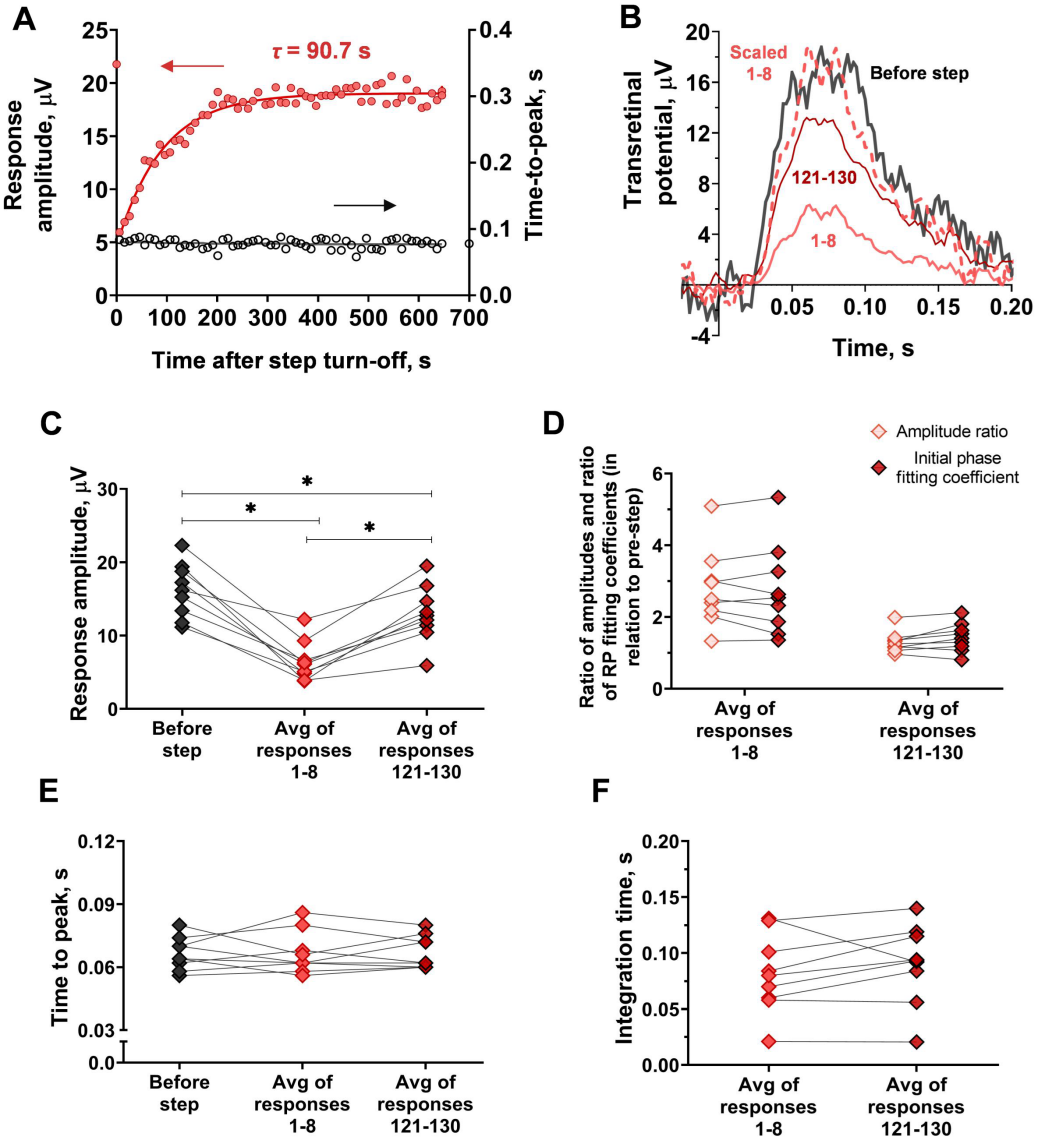
Analysis of response kinetics in relation to ‘adaptation memory’ in red-sensitive frog cones recorded by *ex vivo* ERG. **(A)** Comparison of the sensitivity recovery rate (red circles, left Y-scale) and time-to-peak recovery rate (black circles, right Y-axis). For reference, the point plotted at time zero illustrates the pre-step value. **(B)** Superimposing of selected responses to the same brief test flashes recorded at different time points after step turning-off cut from the recordings presented in Figure 7A. The bold black curve corresponds to pre-step photoresponse; light red curves are the averaged 1st to 8th photoresponses and the dark red curve is the averaged 121st to 130th photoresponses recorded after step turning-off. The dashed curve represents the averaged 1st to 8th response, scaled to coincide with the amplitude of the pre-step. **(C)**–**F)** comparison of several response parameters: response amplitude **(C)**, the ratio of response amplitude and the fitting coefficient for the rising phase (RP fitting coefficient) **(D)**, time-to-peak **(E)** and integration time **(F)**, for pre-step responses, averaged early response after the step turn-off (‘Response 1–8’) and averaged last observable response after step turn-off (‘Response 121–130’). Asterisk ^*^ indicates statistically significant differences between groups (Holm-Sidak test; *p* < 0.05). Sample sizes *n* = 9.

Furthermore, we performed an analysis of response kinetics in relation to the recovery of sensitivity. Due to a relatively high level of noise and low rate of amplitude recovery, it was possible to average the first eight responses after the step of light as a pooled response reflecting the initial stage of recovery and to average the last 10 responses (#121–130) as a pooled response reflecting the late stage of recovery. The results of the comparison of kinetics are shown in Figure 8. One interesting finding is that the time-to-peak, in this case, remained unchanged in contrast to the single rods (repeated measures ANOVA; *p* = 0.94). Integration time also remained stable during amplitude recovery (paired t-test; *p* = 0.24), whereas the initial phase first decreased immediately after the step turning-off and then rose along with the response amplitudes. Specifically, the response undergoes amplitude “scaling” as a result of long-term adaptation. Together, these findings indicate that the mechanisms underlying ‘adaptation memory’ in red-sensitive cones are different from those in rods.

## 4. Discussion

### 4.1. A novel slow adaptation mechanism discovered in rods

Light adaptation is a complex process that involves several different mechanisms resulting in decreased photoreceptor sensitivity during exposure to intense light. In contrast, the dark adaptation that occurs after light turn-off in the case of moderate illumination levels represents the reversal of light adaptation (Firsov et al., 2005) and leads to photoreceptor recovery to a dark-adapted state. The common mechanisms of dark adaptation include relatively fast quenching of activated visual pigment and transducin, together with the restoration of cytoplasmic cGMP concentration to its level in darkness (Chen et al., 2010; Arshavsky and Burns, 2012; Vinberg et al., 2018). These feedback mechanisms depend on the concentration of free cytoplasmic Ca^2+^ which decreases after cationic cGMP-dependent channel closure during illumination. In darkness, as the cell current returns to its dark level, Ca^2+^ concentration and, consequently, the sensitivity also recovers.

Our results (Rotov et al., 2021 and the current study) imply the existence of a novel and previously undescribed mechanism of adaptation in rods with relatively slow kinetics. We showed that even after the full recovery of the cell current to its dark level after turning off the adaptive illumination, the sensitivity still decreased for more than a minute (Figure 1). An exact comparison of the kinetics of recovery of the cell current vs. response amplitude recovery shows that the second process is markedly slower, at least after exposure of the rod to the light exposure steps of moderate and high illumination for more than 20 s (Figure 2). This result proves that there is a different, slow adaptation phase because according to the existing adaptation scheme, the full recovery of the circulating dark current in a rod means return to a balance between PDE and guanylate cyclase activity, that, in turn means the stabilization of intracellular free Ca^2+^ level. Thus, we suppose that after Ca^2+^ concentration becomes stable, rod sensitivity remains decreased as if the cell ‘memorizes’ the preceding adapting illumination for a long time, so we termed this state ‘adaptation memory’. In rods, the light steps of different intensities and durations allowed us to analyze the dependence of cell recovery kinetics from ‘adaptation memory’ on the adaptive light parameters. It appears that the sensitivity time course is highly dependent on the stimulus intensity, resulting in markedly slower recovery as the step brightness increases. In contrast, the light exposure step duration had a minuscule effect on ‘adaptation memory’ for durations >20 s. While low-intensity illumination did not alter the sensitivity recovery rate with prolonged stimulation, the saturating step showed a slight slowdown of this process after prolonging stimulus duration by 4.5 times (from 20 to 90 s; Figure 3).

It is assumed that the time course of sensitivity recovery corresponds to the initial acceleration in brief flash response kinetics and is followed by recovery to the state before light stimulation (Calvert et al., 2002; Astakhova et al., 2008; Chen et al., 2010; Arshavsky and Burns, 2012; Vinberg et al., 2018). A comparison of responses to brief flashes in a dark-adapted state and during recovery of sensitivity after adapting to illumination showed that ‘adaptation memory’ does not induce any change in the steepness of the responses’ initial phase, so no changes in amplification of the phototransduction cascade occur during the adaptation. The changes in response amplitude coincide with the time course of photoresponses, time-to-peak, and integration time that exhibit the accelerated turn-off processes in the ‘adaptation memory’ state (Figure 4). Sensitivity recovery is based on the slowdown of some quenching processes within the cascade. Additionally, the increase in integration time during adaptation should also increase rod sensitivity to prolonged stimuli (several seconds instead of 2 ms). Indeed, in our previous work, we reported a more drastic decrease in amplitude in the ‘adaptation memory’ state for 2 s stimuli compared to 2 ms, while the recovery kinetics remained the same in both cases (Rotov et al., 2021).

Previously, the long-lasting effect of adapting light exposure was thoughtfully studied in mouse rods. First, Krispel et al. showed accelerated quenching of responses to saturating flashes, provided within tens of seconds after the adapting light turn-off and the rod’s dark current full recovery – ‘adaptive acceleration’ (Krispel et al., 2003). The recovery of response kinetics turned out to be dependent on adaptive light intensity and duration. Therefore, we suggest that ‘adaptive acceleration’ may have the same origin as ‘adaptation memory’. Notably, in our studies, it was shown for the first time that adaptive illumination leads not only to the acceleration of photoresponse quenching but also to a drop in sensitivity at the level of subsaturated responses. On the other hand, McKeown and Kraft reported an opposite light-driven effect: a hypersensitization of mouse rods just after background light turn-off or ‘adaptive potentiation’ (McKeown and Kraft, 2014). Our results contradict this work as frog rods show obvious sensitivity loss, however, their increase in saturated and dim-flash response amplitudes may reflect the overall change in rod circulating current instead of hypersensitization. Comparison of our results on frog rods with the studies on mice should take into account that the latter are working with rods, attached to retinal slices in contrast to our isolated cells. Within a slice, the interconnection between different retinal cells may affect the adaptation processes occurring in a rod itself.

### 4.2. Possible mechanisms underlying rod adaptation memory

The discovery of ‘adaptation memory’ proposes the existence of long-lasting modification of the components of the phototransduction cascade after adapting to illumination. This adaptation process appeared to maintain the decreased rod sensitivity long after the full recovery of the dark current level; however, simultaneously, its kinetics is highly light intensity-dependent. Together, this may suggest that the origin of ‘adaptation memory’ is tightly coupled with the decrease in Ca^2+^ cytoplasmic concentration and is realized via mechanisms that are not reverted immediately after recovery of Ca^2+^ levels. For example, Burns et al. reported that ‘adaptive acceleration’ persists in murine rods lacking recovery but is completely abolished in GCAP-deficient animals (Burns et al., 2013), which may indicate the role of the latter in prolonged adaptation along with Ca^2+^-dependent regulation of guanylate cyclase. On the other hand, such Ca^2+^-independent mechanisms, such as the phosphorylation of certain proteins, also appear to be a good explanation for ‘adaptation memory’. Among the non-canonical Ca^2+^-independent mechanisms of sensitivity modulation there is dephosphorylation of cGMP-gated channels by phosphatase due to its activation through insulin-like growth factor-1 receptor (Savchenko et al., 2001) and decreasing channel affinity for cGMP by growth factor receptor-bound protein 14 (Grb14), which result in modulation of time-to-peak and integration time in mouse rod photocurrent (Woodruff et al., 2014). Both insulin-like growth factor-1 receptor and Grb14 are known to be expressed in mammalian rod outer segments (Waldbillig et al., 1987; Rajala et al., 2009), however, nothing is known regarding these targets in frog rods.

Another potential Ca^2+^-independent pathway for modulating response decay, as a consequence of its sensitivity, is the phosphorylation of the rhodopsin kinases. It was shown that for the effective dark adaptation in rods, the phosphorylation of G protein-coupled receptor kinase 1 (GRK1) is of critical importance and this phosphorylation is cAMP-dependent (Kolesnikov et al., 2020). Previously we also demonstrated that an artificial increase of intracellular cAMP in frog isolated rods results in slowing down response turn-off (Astakhova et al., 2012), so adapting illumination could putatively decrease the cAMP level, and if this decrease persists for several tens of seconds it could account for the temporary drop in sensitivity originating from accelerating response quenching. All the more so, GRK1 was demonstrated to be expressed not only in mammalian rods but also in frog rods (Osawa and Weiss, 2012).

It is important to mention the possibility that rod post-illumination adaptation originates from the activity of free opsin that appears after bleaching and its residual ability to excite the phototransduction cascade (Fain et al., 2001). However, several factors count against its significant role in ‘adaptation memory’. First, the estimated bleaching effect from adapting light used in our study was very low, not exceeding 0.08% (for the step of highest intensity and duration 90 s used), while any detectable kinetics acceleration is reported for bleaches higher than 5% (Nymark et al., 2012). Second, it is known that the adapting effect of free opsin is persistent and rod photoresponse kinetics can only be recovered after applying the exogenous chromophore to restore the pigment (Cornwall and Fain, 1994; Jones et al., 1996). In our case, the restoration of response kinetics within several tens of seconds after adapting the light turn-off is observed without any chromophore supply. Third, the photolysis rate of amphibian rhodopsin is relatively slow and it usually takes more than 20 min to fully decay to opsin and retinal after bleaching (Golobokova and Govardovskii, 2006). Therefore, one should expect that for the first several minutes after the adapting illumination, the concentration of free opsin (as well as meta-products possessing residual activity too) should rise up and so cause progressive response acceleration over time, which is contrary to our observations. Thus, it seems that free opsin does not play any significant role in ‘adaptation memory’.

### 4.3. Cones demonstrate a slow adaptation phase only as a part of the retinal tissue

In the second part of our study, we found that the cones of fish and frogs have a slow phase of sensitivity recovery after steps of bright light that, at first glance, resembles the phenomenon observed in single frog rods. However, cones are known as the photoreceptors that have traded their sensitivity for fast kinetics during evolution (Lamb, 2013), thus, their basic phototransduction turn-off processes – visual pigment and transducin inactivation, together with cGMP and Ca^2+^ turnover rate – are approximately an order of magnitude faster than those of rods (Astakhova et al., 2015). From this point, the cones’ recovery from any assumed ‘adaptation memory’ state is expected to occur at a higher rate than that of rods.

In trying to compare the kinetic parameters of ‘adaptation memory’ in frog rods and isolated *C. gibelio* cones, we failed to discover any signs of slow adaptation that could be attributed to, for example, the long-lasting colored negative afterimages (Figure 5). Adapting light of moderate intensity (approximately 0.8% pigment bleach during a 40 s exposure step) decreased the sensitivity of isolated cones for a short time (several seconds), thus, recovery occurred approximately an order of magnitude faster than that in the rods. The use of high-intensity illumination levels leads to a higher bleaching degree of the cone visual pigment (> 10%) and an irreversible drop in sensitivity. In this state, the variability in response amplitude originating from signal noise has the same order of magnitude as its assumed decrease due to ‘adaptation memory’, which makes any of its possible effects on cell sensitivity negligible. Thus, there is no evidence of any significant slow adaptation processes at the level of isolated single cones.

If the visual pigment bleaching results in irreversibly lowered sensitivity, the use of isolated retinal tissue may solve this problem. It is well known that cones can regenerate their visual pigment via the specific pathway involving Müller glial cells (Wang et al., 2009; Kaylor et al., 2013), therefore, we expected them to restore it during the adapting light step. Then more visual pigment would remain after bright illumination, making it possible to produce a more drastic decrease in sensitivity so that cone ‘adaptation memory’ could be reliably analyzed. We observed an extremely slow recovery of response amplitudes, well-fitted with a single exponential function (Figures 6, 7) after exposure of the retina to highly bleaching light exposure steps (from 30 to 90% if no regeneration occurred) that is in line with previous studies (Wang et al., 2009; Wang and Kefalov, 2009). However, the estimation of pigment bleaching showed that due to the continuous regeneration via Müller cells, the actual post-step bleach amount range was 6–34%. We showed that the kinetics of this process did not depend on adaptive step duration, while it slightly increased with an increase in its intensity. Such properties do not match with rod ‘adaptation memory’, but can be explained in terms of pigment regeneration, since the rate of 11-cis-retinol production by Müller cells (Sato and Kefalov, 2016) may adapt to the rate of pigment bleaching by a light stimulus.

Analysis of response kinetics confirmed that slow recovery from ‘adaptation memory’ in cones is based on a different mechanism than those in rods because the integration time and time-to-peak of the flash response recovery in rods, after the adapting step, do not change with bright steps of light in cones (Figure 8). The change in the initial phase steepness coincides with amplitude recovery, which indicates that responses in the adaptive state are being scaled instead of accelerated. Such a situation can occur when a sensitivity drop is caused by a decrease in the availability of the visual pigment, that is, by bleaching. Still, this result is quite unexpected since one would expect the response kinetics to accelerate due to the adapting effect of free opsin (see subsection 4.2 and also Jones et al., 1993; Cornwall et al., 1995). Indeed, the responses used for kinetics analysis were exposed to adapting light that bleached 17 ± 4% of the visual pigment, which seems to be enough to cause significant adaptation. Moreover, the level of desensitization after the light step can only be reliably explained if free opsin adaptation impact is taken into account (see Supplementary Figure S1). As cone pigments decay several times faster than rhodopsin without the formation of stable meta-products (Golobokova and Govardovskii, 2006), there should be a significant amount of free opsin at the time of adapting step turn-off. Taken together, we have shown that in the presence of a constant chromophore supply, the cone response quenching acceleration is abolished, at least for the bleach amounts not exceeding 20%.

It should be noted that in the living eye, there would also be an impact on regeneration from the retinal pigment epithelium (Wang et al., 2009). In addition, the loss of the photoreceptor extracellular matrix in the isolated retinal preparation must be considered along with rapid perfusion, which may wash away Müller cells-derived chromophore and thus further reduce the rate of pigment regeneration. Therefore, the overall rate of sensitivity recovery is expected to be higher than that reported in the current study.

### 4.4. ‘Adaptation memory’ of different origin: possible connection with visual afterimages

The pattern of rod sensitivity recovery after the steps of light strongly resembles the phenomenology of visual afterimages. After looking at a bright source of light and then closing the eyes, one can see that the light source slowly fades away. This is called the positive afterimage, which lasts for a short time (Craik, 1940) and when it fades completely, its dark copy on the black background appears and this is called the negative afterimage. If the light source is colored, then the afterimage has complementary colors. In humans, the disappearance of afterimages can take up to 100–300 s for moderate and brief light stimuli (Brindley, 1959) and up to 15–20 min in case of brief and very bright stimuli (Brindley, 1962). The origin of visual afterimages has been debated for a long time, and the following question remain: are they located in the retina, or do they result from central mechanisms?

The described phenomena of ‘adaptation memory’ provides a post-illumination long-lasting state of reduced sensitivity that perfectly fits the negative image. It should be noted that the origin of visual afterimages might also lie within neural networks of the inner retina if they can maintain their excited state long enough. However, these two original hypotheses do not contradict each other (Loomis, 1972; Virsu and Laurinen, 1977).

It is important to study a visual analyzer as a complex system, not only at the level of its single units. Therefore, considering the photoreceptors in the living eye, one should also consider the existence of other slow dark adaptation mechanisms that remain outside the scope of this study. First, the translocation of transducin, arrestin, and recoverin occurs between the outer and inner segments of a rod under continuous background illumination (Sokolov et al., 2002; Strissel et al., 2005; Frederiksen et al., 2021). Further sensitivity reduction could be achieved by the retinomotor movement of melanin granules spreading from the bodies of pigment epithelium cells to their processes (Miller and Snyder, 1972; Easter and Macy, 1978). Even at the level of a retinal slice, the rod responses demonstrate a hypersensitization instead of ‘adaptation memory’ that additionally highlights the ambiguity of the extrapolation of physiological phenomena from isolated cells to living organisms.

The situation becomes more complicated for colored afterimages that are expected to be connected with cone pathways. We demonstrated the slow recovery of cone sensitivity after highly bleached light steps via the inner retinal pigment regeneration mechanism. The kinetics of this process fits the negative afterimage phenomenology well; moreover, it can be realized only in cones, because of their fast visual pigment post-bleach decay and inclusion in the visual cycle. Rods, even those with access to the retinal pigment epithelium, necessary for their pigment regeneration, would demonstrate much slower kinetics with their stable post-bleach metaproducts (Golobokova and Govardovskii, 2006). This study has described similar processes of ‘adaptation memory’ in both rods and cones, which are based on fundamentally different mechanisms: biochemical adaptation in the phototransduction cascade in rods, and the photochemical visual pigment cycling in cones.

## Data availability statement

The raw data supporting the conclusions of this article will be made available by the authors, without undue reservation.

## Ethics statement

The animal study was reviewed and approved by Bioethics Committee of the IEPHB RAS.

## Author contributions

DN: performed electrophysiology recordings and formal analysis and wrote the original draft. LA and AR: conceptualization, performed electrophysiology recordings, and wrote the original draft. LA: funding acquisition. MN: performed electrophysiology recordings and formal analysis. All authors contributed to the article and approved the submitted version.

## Funding

This research was funded by the Russian Science Foundation, Project no. 22–25-00591.

## Conflict of interest

The authors declare that the research was conducted in the absence of any commercial or financial relationships that could be construed as a potential conflict of interest.

## Publisher’s note

All claims expressed in this article are solely those of the authors and do not necessarily represent those of their affiliated organizations, or those of the publisher, the editors and the reviewers. Any product that may be evaluated in this article, or claim that may be made by its manufacturer, is not guaranteed or endorsed by the publisher.

## References

[ref1] Arshavsky V. Y. Burns M. E. (2012). Photoreceptor signaling: supporting vision across a wide range of light intensities. J. Biol. Chem. 287, 1620–1626. doi: 10.1074/jbc.R111.305243 22074925 PMC3265842

[ref2] Astakhova L. A. Firsov M. L. Govardovskii V. I. (2008). Kinetics of turn-offs of frog rod phototransduction cascade. J. Gen. Physiol. 132, 587–604. doi: 10.1085/jgp.200810034 18955597 PMC2571975

[ref3] Astakhova L. A. Firsov M. L. Govardovskii V. I. (2015). Activation and quenching of the phototransduction cascade in retinal cones as inferred from electrophysiology and mathematical modeling. Mol. Vis. 21, 244–263. 25866462 PMC4392649

[ref4] Astakhova L. A. Samoiliuk E. V. Govardovskii V. I. Firsov M. L. (2012). cAMP controls rod photoreceptor sensitivity via multiple targets in the phototransduction cascade. J. Gen. Physiol. 140, 421–433. doi: 10.1085/jgp.201210811 23008435 PMC3457688

[ref5] Baylor D. A. Lamb T. D. Yau K. W. (1979). The membrane current of single rod outer segments. J. Physiol. 288, 58–611. doi: 10.1113/jphysiol.1979.sp012715 PMC1281446 112242

[ref6] Brindley G. S. (1959). The discrimination of after-images. J. Physiol. 147:194. doi: 10.1113/jphysiol.1959.sp006234 13673397 PMC1357015

[ref7] Brindley G. S. (1962). Two new properties of foveal after-images and a photochemical hypothesis to explain them. J. Physiol. 164:168. doi: 10.1113/jphysiol.1962.sp007011 14015499 PMC1359294

[ref8] Burns M. Gross O. Krispel C. (2013). Adaptive acceleration in mouse rods is mediated by slow feedback via guanylate cyclase activating proteins. Investig. Ophthalmol. Vis. Sci. 54:2457.23482465

[ref9] Calvert P. D. Govardovskii V. I. Arshavsky V. Y. Makino C. L. (2002). Two temporal phases of light adaptation in retinal rods. J. Gen. Physiol. 119, 129–145. doi: 10.1085/jgp.119.2.129 11815664 PMC2233805

[ref10] Chen J. Woodruff M. L. Wang T. Concepcion F. A. Tranchina D. Fain G. L. (2010). Channel modulation and the mechanism of light adaptation in mouse rods. J. Neurosci. 30, 16232–16240. doi: 10.1523/JNEUROSCI.2868-10.2010 21123569 PMC3010974

[ref11] Cornwall M. C. Fain G. L. (1994). Bleached pigment activates transduction in isolated rods of the salamander retina. J. Physiol. 480, 261–279. doi: 10.1113/jphysiol.1994.sp020358 7532713 PMC1155844

[ref12] Cornwall M. C. Matthews H. R. Crouch R. K. Fain G. L. (1995). Bleached pigment activates transduction in salamander cones. J. Gen. Physiol. 106, 543–557. doi: 10.1085/jgp.106.3.543 8786347 PMC2229273

[ref13] Craik K. J. W. (1940). Origin of visual after-images. Nature 145:512.

[ref14] Easter S. S. Macy A. (1978). Local control of retinomotor activity in the fish retina. Vis. Res. 18, 937–942. doi: 10.1016/0042-6989(78)90021-4 706169

[ref15] Fain G. L. Matthews H. R. Cornwall M. C. Koutalos Y. (2001). Adaptation in vertebrate photoreceptors. Physiol. Rev. 81, 117–151. doi: 10.1016/0042-6989(90)90013-B 11152756

[ref16] Firsov M. L. Govardovskii V. I. (2001). Photoreceptor light adaptation: meanings and mechanisms. Sens. Syst. 15, 101–113. (In Russ)

[ref17] Firsov M. L. Kolesnikov A. V. Golobokova E. Y. Govardovskii V. I. (2005). Two realms of dark adaptation. Vis. Res. 45, 147–151. doi: 10.1016/j.visres.2004.08.005 15581916

[ref18] Frederiksen R. Morshedian A. Tripathy S. A. Xu T. Travis G. H. Fain G. L.. (2021). Rod photoreceptors avoid saturation in bright light by the movement of the G protein transducin. J. Neurosci. 41, 3320–3330. doi: 10.1523/JNEUROSCI.2817-20.202 33593858 PMC8051685

[ref19] Goldstein E. B. (1970). Cone pigment regeneration in the isolated frog retina. Vis. Res. 10, 1065–1068. doi: 10.1016/0042-6989(70)90082-9 5492790

[ref20] Golobokova E. Y. Govardovskii V. I. (2006). Late stages of visual pigment photolysis in situ: cones vs. rods. Vis. Res. 46, 2287–2297. doi: 10.1016/j.visres.2005.12.017 16473387

[ref21] Govardovskii V. I. Fyhrquist N. Reuter T. Kuzmin D. G. Donner K. (2000). In search of the visual pigment template. Vis. Neurosci. 17, 509–528. doi: 10.1017/S0952523800174036 11016572

[ref22] Hood D. C. Hock P. A. (1973). Recovery of cone receptor activity in the frog's isolated retina. Vis. Res. 13, 1943–1951. doi: 10.1016/0042-6989(73)90065-5 4542882

[ref23] Jones G. J. Cornwall M. C. Fain G. L. (1996). Equivalence of background and bleaching desensitization in isolated rod photoreceptors of the larval tiger salamander. J. Physiol. 108, 333–340. doi: 10.1085/jgp.108.4.333 PMC2229334 8894981

[ref24] Jones G. J. Fein A. MacNichol E. F. Jr. Cornwall M. C. (1993). Visual pigment bleaching in isolated salamander retinal cones. Microspectrophotometry and light adaptation. J. Gen. Physiol. 102, 483–502. doi: 10.1085/jgp.102.3.483 8245820 PMC2229157

[ref25] Kaylor J. J. Yuan Q. Cook J. Sarfare S. Makshanoff J. Miu A.. (2013). Identification of DES1 as a vitamin a isomerase in Müller glial cells of the retina. Nat. Chem. Biol. 9, 30–36. doi: 10.1038/nchembio 23143414 PMC3522777

[ref26] Kefalov V. J. Estevez M. E. Kono M. Goletz P. W. Crouch R. K. Cornwall M. C.. (2005). Breaking the covalent bond – a pigment property that contributes to desensitization in cones. Neuron 46, 879–890. doi: 10.1016/j.neuron.2005.05.009 15953417 PMC2885911

[ref27] Kolesnikov A. V. Chrispell J. D. Osawa S. Kefalov V. J. Weiss E. R. (2020). Phosphorylation at serine 21 in G protein-coupled receptor kinase 1 (GRK1) is required for normal kinetics of dark adaptation in rod but not cone photoreceptors. FASEB J. 34:2677. doi: 10.1096/fj.201902535R 31908030 PMC7043924

[ref28] Krispel C. M. Chen C. K. Simon M. I. Burns M. E. (2003). Novel form of adaptation in mouse retinal rods speeds recovery of phototransduction. J. Gen. Physiol. 122, 703–712. doi: 10.1085/jgp.200308938 14610022 PMC2229593

[ref29] Lamb T. D. (2013). Evolution of phototransduction, vertebrate photoreceptors and retina. Prog. Retin. Eye Res. 36, 52–119. doi: 10.1016/j.preteyeres.2013.06.001 23792002

[ref30] Loomis J. M. (1972). The photopigment bleaching hypothesis of complementary after-images: a psychophysical test. Vis. Res. 12, 1587–1594. doi: 10.1016/0042-6989(72)90031-4 5078784

[ref31] McKeown A. S. Kraft T. W. (2014). Adaptive potentiation in rod photoreceptors after light exposure. J. Gen. Physiol. 143, 733–743. doi: 10.1085/jgp.201411163 24821966 PMC4035749

[ref32] Miller W. H. Snyder A. W. (1972). Optical function of myoids. Vis. Res. 12, 1841–1848. doi: 10.1016/0042-6989(72)90074-0 4562458

[ref33] Nymark S. Frederiksen R. Woodruff M. L. Cornwall M. C. Fain G. L. (2012). Bleaching of mouse rods: microspectrophotometry and suction-electrode recording. J. Physiol. 590, 2353–2364. doi: 10.1113/jphysiol.2012.228627 22451436 PMC3424757

[ref34] Osawa S. Weiss E. R. (2012). A tale of two kinases in rods and cones. Adv. Exp. Med. Biol. 821–827, 821–827. doi: 10.1007/978-1-4614-0631-0_105 PMC3632502 22183412

[ref35] Rajala A. Daly R. J. Tanito M. Allen D. T. Holt L. J. Lobanova E. S.. (2009). Growth factor receptor-bound protein 14 undergoes light-dependent intracellular translocation in rod photoreceptors: functional role in retinal insulin receptor activation. Biochemistry 48, 5563–5572. doi: 10.1021/bi9000062 19438210 PMC2763493

[ref36] Röhlich P. Szél Á. (2000). Photoreceptor cells in the Xenopus retina. Microsc. Res. Tech. 50, 327–337. doi: 10.1002/1097-0029(20000901)50:5<327::AID-JEMT2>3.0.CO;2-P 10941169

[ref37] Rotov A. Y. Astakhova L. A. Firsov M. L. Govardovskii V. I. (2017). Origins of the phototransduction delay as inferred from stochastic and deterministic simulation of the amplification cascade. Mol. Vis. 23:416. 28744093 PMC5509446

[ref38] Rotov A. Y. Astakhova L. A. Firsov M. L. Govardovskii V. I. (2021). Light adaptation of retinal rods, adaptation memory, and afterimages. Neurosci. Behav. Physiol. 51, 116–122. doi: 10.1007/s11055-020-01046-2

[ref39] Sato S. Kefalov V. J. (2016). Cis retinol oxidation regulates photoreceptor access to the retina visual cycle and cone pigment regeneration. J. Physiol. 594, 6753–6765. doi: 10.1113/JP272831 27385534 PMC5108915

[ref40] Savchenko A. Kraft T. W. Molokanova E. Kramer R. H. (2001). Growth factors regulate phototransduction in retinal rods by modulating cyclic nucleotide-gated channels through dephosphorylation of a specific tyrosine residue. Proc. Natl. Acad. Sci. U. S. A. 98, 5880–5885. doi: 10.1073/pnas.101524998 11320223 PMC33307

[ref41] Sokolov M. Lyubarsky A. L. Strissel K. J. Savchenko A. B. Govardovskii V. I. Pugh E. N. Jr.. (2002). Massive, light-driven translocation of transducin between the two major compartments of rod cells: a novel mechanism of light adaptation. Neuron 34, 95–106. doi: 10.1016/s0896-6273(02)00636-0 11931744

[ref42] Strissel K. J. Lishko P. V. Trieu L. H. Kennedy M. J. Hurley J. B. Arshavsky V. Y. (2005). Recoverin undergoes light-dependent intracellular translocation in rod photoreceptors. J. Biol. Chem. 280, 29250–29255. doi: 10.1074/jbc.M501789200 15961391

[ref43] Vinberg F. Chen J. Kefalov V. J. (2018). Regulation of calcium homeostasis in the outer segments of rod and cone photoreceptors. Prog. Retin. Eye Res. 67, 87–101. doi: 10.1016/j.preteyeres.2018.06.001 29883715 PMC6235702

[ref44] Virsu V. Laurinen P. (1977). Long-lasting afterimages caused by neural adaptation. Vis. Res. 17, 853–860. doi: 10.1016/0042-6989(77)90129-8 898691

[ref45] Waldbillig R. J. Fletcher R. T. Chader G. J. Rajagopalan S. Rodrigues M. LeRoith D. (1987). Retinal insulin receptors. 2. Characterization and insulin-induced tyrosine kinase activity in bovine retinal rod outer segments. Exp. Eye Res. 45, 837–844. doi: 10.1016/S0014-4835(87)80100-8 3322853

[ref46] Wang J. S. Estevez M. E. Cornwall M. C. Kefalov V. J. (2009). Intra-retinal visual cycle required for rapid and complete cone dark adaptation. Nat. Neurosci. 12, 295–302. doi: 10.1038/nn.225 19182795 PMC2707787

[ref47] Wang J. S. Kefalov V. J. (2009). An alternative pathway mediates the mouse and human cone visual cycle. Curr. Biol. 19, 1665–1669. doi: 10.1016/j.cub.2009.07.05 19781940 PMC2762012

[ref48] Wolf G. (2004). The visual cycle of the cone photoreceptors of the retina. Nutr. Rev. 62:283. doi: 10.1111/j.1753-4887.2004.tb00053.x 15384919

[ref49] Woodruff M. L. Janisch K. M. Peshenko I. V. Dizhoor A. M. Tsang S. H. Fain G. L. (2008). Modulation of phosphodiesterase6 turnoff during background illumination in mouse rod photoreceptors. J. Neurosci. 28, 2064–2074. doi: 10.1523/JNEUROSCI.2973-07.2008 18305241 PMC2750778

[ref50] Woodruff M. L. Rajala A. Fain G. L. Rajala R. V. (2014). Modulation of mouse rod photoreceptor responses by Grb14 protein. J. Biol. Chem. 289, 358–364. doi: 10.1074/jbc.M113.517045 24273167 PMC3879558

