# Supplementary material for: Adaptation memory in photoreceptors: different mechanisms in rods and cones
Source: Front Mol Neurosci. 2023 Apr 24;16:1135088. doi: 10.3389/fnmol.2023.1135088 (PMC10165083; doi:10.3389/fnmol.2023.1135088)
Supplement: Supplementary file 1 [file Data_Sheet_1.docx]

***Supplementary Material***

Adaptation memory in photoreceptors: different mechanisms in rods and cones

**Darya A. Nikolaeva^1^, Maria A. Nekrasova^1^, Alexander Yu. Rotov^1^, Luba A. Astakhova^1*^**

^1^Laboratory of Evolution of the Sense Organs, Sechenov Institute of Evolutionary Physiology and Biochemistry RAS, 194223 St. Petersburg, Russia

*** Correspondence:**Luba A. Astakhova
[lubkins@yandex.ru](mailto:lubkins@yandex.ru)

**Calculation of visual pigment bleaching**

First, we measured the light energy of the stimulating light and further converted it to the photon flux density based on the known absorbance spectrum of OPT-301 optosensor and emission spectra of our LEDs (combined with proper cut-off filters). Then, the stimulus intensity was recalculated from number of photons per µm^2^ into number of photoisomerizations in a unit cell volume (R*/μm^3^) per millisecond of illumination time. This value is usually defined as “effective light intensity” I_e_(λ) and can be calculated by the following formula (see, for example, Astakhova et al., 2015):

| I_e_(λ) = 2.303*I(λ)*q*f*a_max_*S(λ), | (1) |
| --- | --- |

Here, I(λ) is the light intensity expressed as the photons/µm^2^/ms, q = 0.67 is the quantum yield of the visual pigment bleaching (Dartnall, 1972), a_max_ is the density of the corresponding visual pigment (in µm^-1^), and f is a correcting factor for light polarization. The last two values were estimated by microspectrophotometry and appeared to be 0.014 µm^-1^ and 0.62 for frog rods, 0.012 µm^-1^ and 0.64 for *C. gibelio* LWS cones and 0.008 µm^-1^ and 0.64 for frog LWS cones. S(λ) is the relative spectral sensitivity of the visual pigment to the stimulus emission. To define these values we calculated the overlap of the visual pigment absorbance spectra (defined from microspectrophotometric measurements fitted with standard template, see Govardovskii et al., 2000) with the emission spectrum of the green LED and determined the integral of the product normalized on the LED emission.

In the case of isolated rods and cones that lack any chromophore supply from either the pigment epithelium or Muller cells, the fraction of bleached pigment F was calculated as follows:

| F = 1 – exp(-I_e_(λ)*T/N), | (2) |
| --- | --- |

Here, T is the duration of light exposure, N is the number of visual pigment molecules per μm^3^ of cell volume. The latter was estimated from a typical concentration of visual pigment within the photoreceptor outer segment of 3 mM (Harosi, 1975) with the correction for the presence of free opsin in cones in their dark-adapted state. We assumed it to be 32% for *C. gibelio* and 10% for frog LWS cones, respectively (according to data on goldfish and tiger salamander, see Luo et al., 2020 and Kefalov et al., 2005).

In the case of cones from isolated retina preparations, the estimation is more complicated, since the chromophore supply from Muller cells affects sensitivity as well as the bleaching light. Moreover, our protocol involves exposure of the retina to a continuous background light in order to suppress rod activity. Unlike salamander cones (studied by Kefalov et al., 2005) frog cones cannot provide robust responses being isolated from the retina, so the calibration experiments are restricted to the ERG recordings and, therefore, limited to provide accurate estimation of bleaching. Still, to make at least a rough estimation of the amount of pigment bleached by background light during our experiments, we recorded the recovery of cone response after turn-off of prolonged exposure to rod-suppressing illumination (lasting at least 90 min, so the bleach of the rod pigment would be over 90% and their input in ERG would be negligible) suggesting that the visual pigment returned to the dark-adapted level within 10-15 min in darkness. Then we used the relation between the amount of bleached pigment and the decrease in sensitivity derived previously for salamander LWS cones (see Jones et al., 1993; Kefalov et al., 2005):

| S_BG_/S = (1 - F)/(1 + kF), | (3) |
| --- | --- |

Here S and S_BG_ are the flash sensitivities of cones after dark adaptation (and on the assumption of pigment recovery equal to 100%) and under the rod-suppressing background, respectively, and k is a certain constant calculated to be 8.6 for salamander LWS cones. As we stimulated retinal preparations with half-saturating flashes (for background-adapted state), the photoreceptor sensitivity shift S/S_BG_ can roughly be estimated as a ratio between response amplitudes to the same flash under different light conditions. Using the relation for salamander cone we calculated the bleach caused by rod-suppressing background to be approximately 1.7% and take it into account together with the abovementioned 10% of free opsin to calculate the amount of pigment in a LWS cone under background illumination (and subsequently, its proportion bleached by further stimulation). In the next step, to improve the accuracy of our calculations, we performed a set of calibration experiments to obtain a relation between the amount of bleached pigment and the decrease in frog LWS cones sensitivity. We exposed the retina to short flashes that bleached approximately 8, 26, 35 and 50% of pigment and compared the response amplitudes before and immediately after the flash. The results are depicted in Fig. S1, where the experimental values of desensitization are best-fitted with the equation (3) with k = 5.5. Using this parameter, we were able to estimate the amount of bleaching after adapting light steps in our ‘adaptation memory’ investigations.

References:

1. Astakhova, L. A., Firsov, M. L., and Govardovskii, V. I. (2015). Activation and quenching of the phototransduction cascade in retinal cones as inferred from electrophysiology and mathematical modeling. *Mol. Vis.* 21, 244–263. PMID: 25866462
2. Dartnall, H. J. A. (1972). Photosensitivity. *Photochemistry of vision.* *Handbook of Sensory Physiology.* Berlin, Heidelberg: Springer, 122–145. doi: 10.1007/978-3-642-65066-6_4
3. Govardovskii, V. I., Fyhrquist, N., Reuter, T., Kuzmin, D. G., and Donner, K. (2000). In search of the visual pigment template. *Vis. Neurosci.* 17, 509–528. doi: 10.1017/S0952523800174036
4. Hárosi, F. I. (1975). Absorption spectra and linear dichroism of some amphibian photoreceptors. *J. Gen. Physiol.* 66, 357-382. doi: 10.1085/jgp.66.3.357
5. Jones, G. J., Cornwall, M. C., and Fain, G. L. (1996). Equivalence of background and bleaching desensitization in isolated rod photoreceptors of the larval tiger salamander. *J. Gen. Physiol.* 108, 333–340. doi: 10.1085/jgp.108.4.333
6. Kefalov, V. J., Estevez, M. E., Kono, M., Goletz, P. W., Crouch, R. K., Cornwall, M. C., and Yau, K. W. (2005). Breaking the covalent bond – a pigment property that contributes to desensitization in cones. *Neuron* 46, 879–890. doi: 10.1016/j.neuron.2005.05.009
7. Luo, D. G., Silverman, D., Frederiksen, R., Adhikari, R., Cao, L. H., Oatis, J. E., Kono, M., Cornwall, M. C., and Yau, K. W. (2020). Apo-opsin and its dark constitutive activity across retinal cone subtypes. *Curr. Biol.* 30, 4921-4931. doi: 10.1016/j.cub.2020.09.062

**
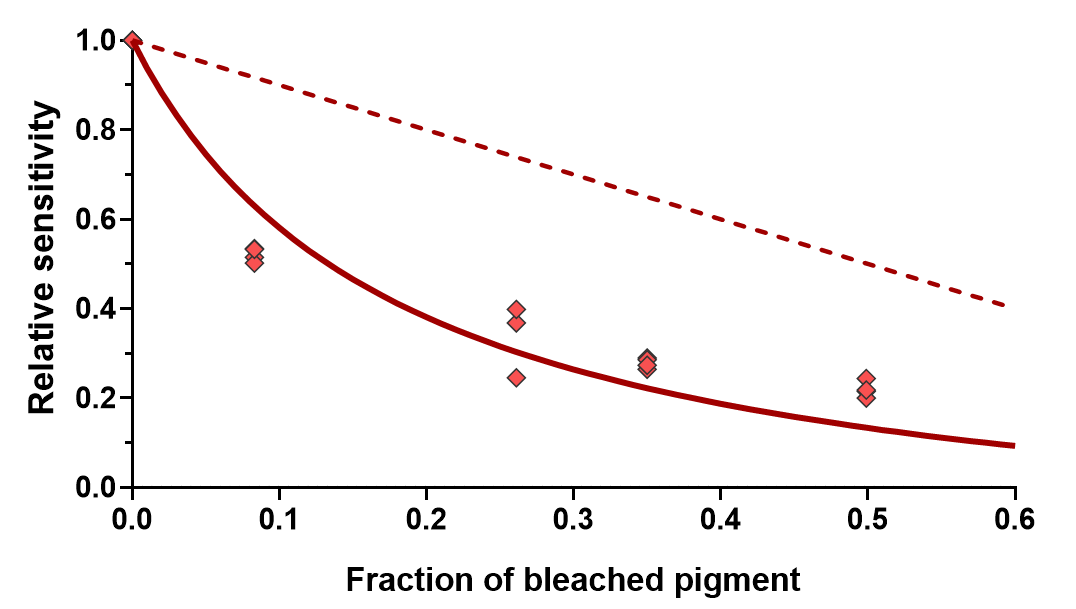
**

**Figure S1.** Calibration experiments to determine the relation between fraction of the bleached pigment and decrease in sensitivity to test flashes in frog LWS cones. Combined data from 4 preparations. Solid line – the best fit of the experimental data with equation (3) with constant k = 5.5. Dashed line – relation between the fraction of bleached pigment and decrease in sensitivity that takes into account only the drop in pigment content but not the residual activity of free opsin (determined by equation (3) lacking the denominator).


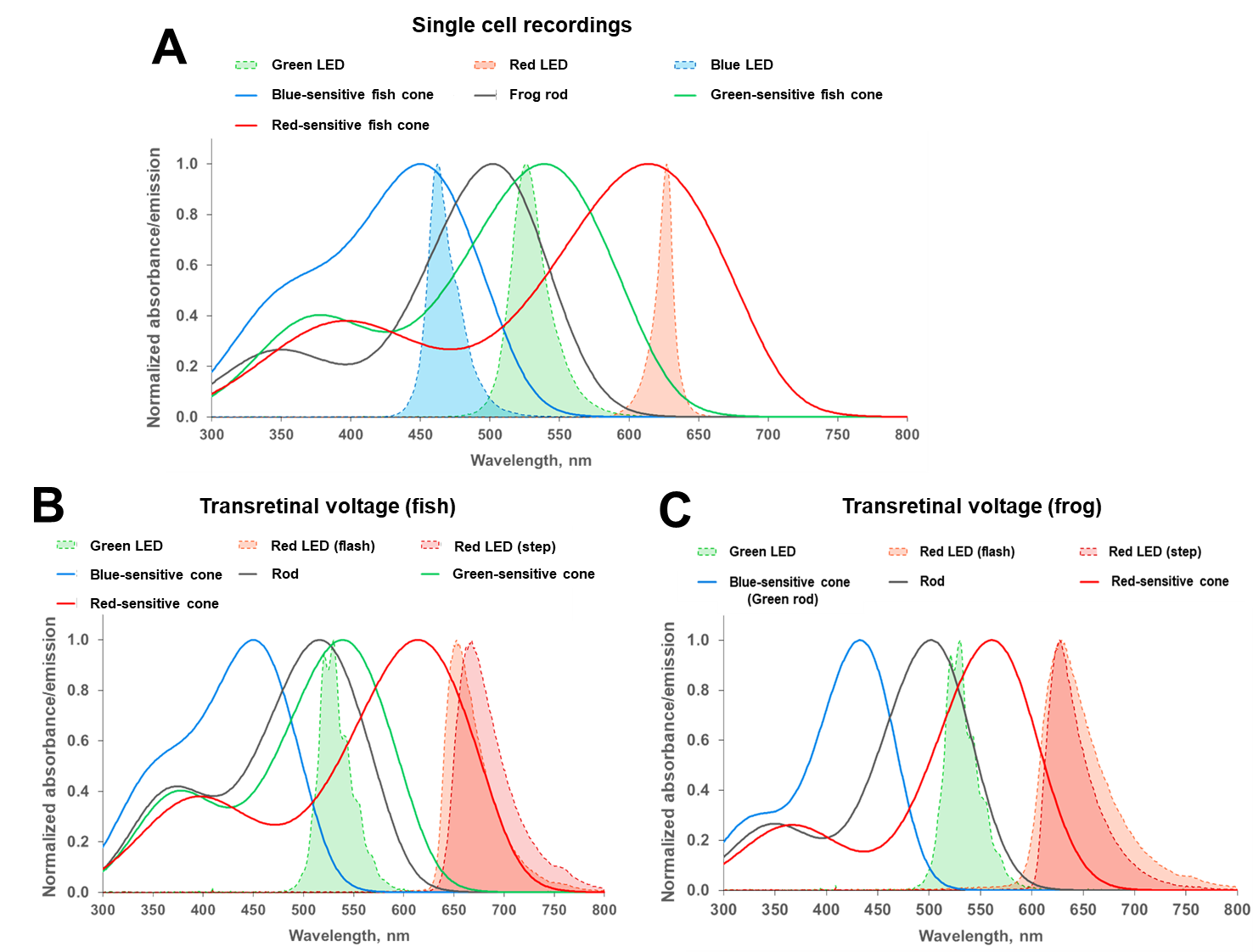


**Figure S2.** Sensitivities of the photoreceptors to light stimuli used in the electrophysiological experiments. The absorbance spectra are based on standard template fits for marsh frog and goldfish visual pigments (see Govardovskii et al., 2000). Filled areas show the emission spectra of LEDs used in either suction pipette (A) or transretinal voltage (B, C) recording setups. Emission spectra measurements were performed using USB4000 spectrometer (Ocean Optics, USA). Red stimuli from panels B and C represent emission of white LEDs, supported with different long-wavelength pass filters. Spectra of UV-sensitive cones are not shown.

For a suction electrode, LEDs peaking at 460 nm (half bandwidth 454 – 477 nm), 525 nm (513 – 541 nm) and 630 nm (620 – 633 nm) were used. For the transretinal recording setup we used the green LED peaking at 530 nm (half bandwidth 514 – 546 nm) together with two white LEDs combined with red cut-off filters. Since frog and *C. gibelio* LWS cones have different spectral sensitivity, we used different stimuli for them: peaking at 627 (613 – 653 nm) and 629 nm (608 – 666 nm) in case of frog; peaking at 653 (640 – 680 nm) and 668 nm (650 – 699 nm) in case of fish.


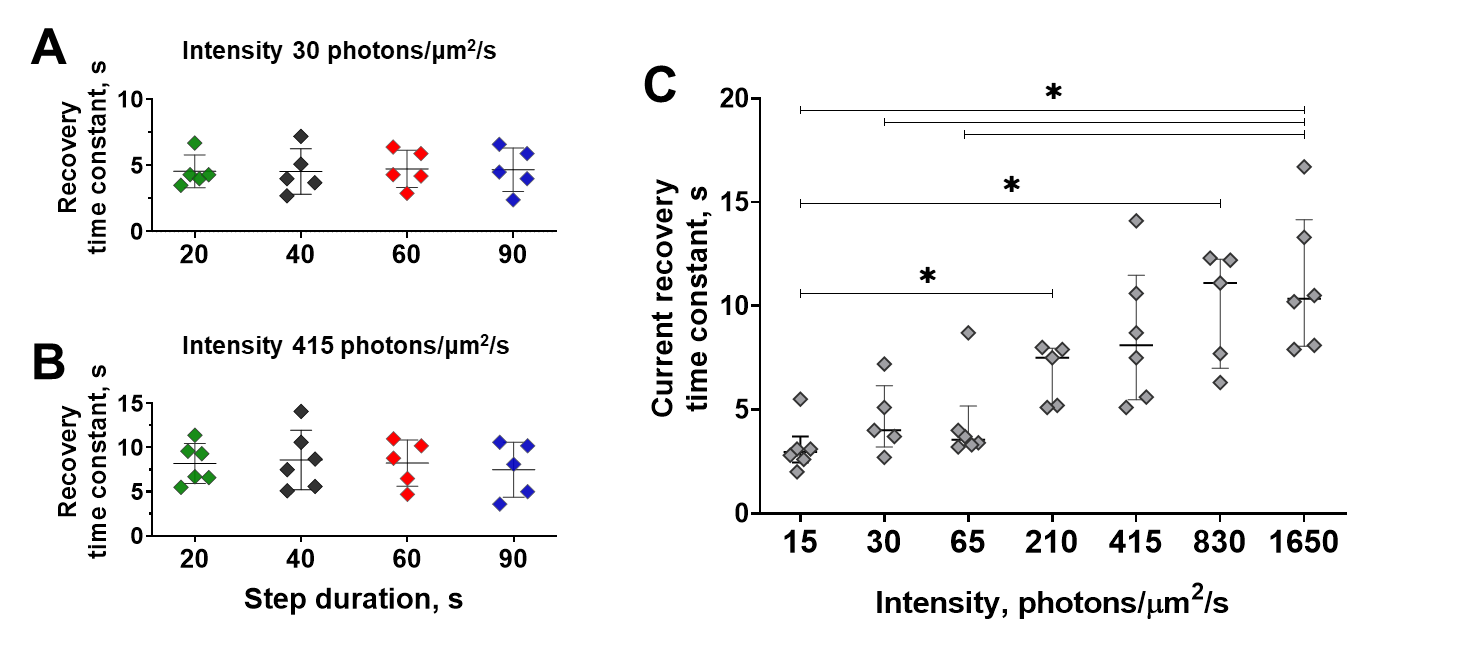


**Figure S3.**  Comparison of the rate of current recovery after light steps of increasing duration and intensity in frog rods. The recovery time constant refers to a single-exponential fit of decreasing phase of photoresponse after light step turn-off. Changes in step duration do not affect the time course of current recovery for both moderate (A) and saturating (B) intensities (Welch’s ANOVA test, p=0.996 and 0.960, respectively). On the other hand, the increase in 40 s stimulus intensity leads to a significant slowdown in current recovery (C), according to Welch’s ANOVA test, p < 0.0001. The linear regression slope for this dependence is also significant (slope coefficient = 0.0045 photons^-1^•μm^2^•s^2^, p < 0.01, R^2^ = 0.8). * – statistically significant differences between groups (Dunnett's multiple comparisons test, p < 0.05). Sample sizes n = 5-6. Bars represent mean ± SD.


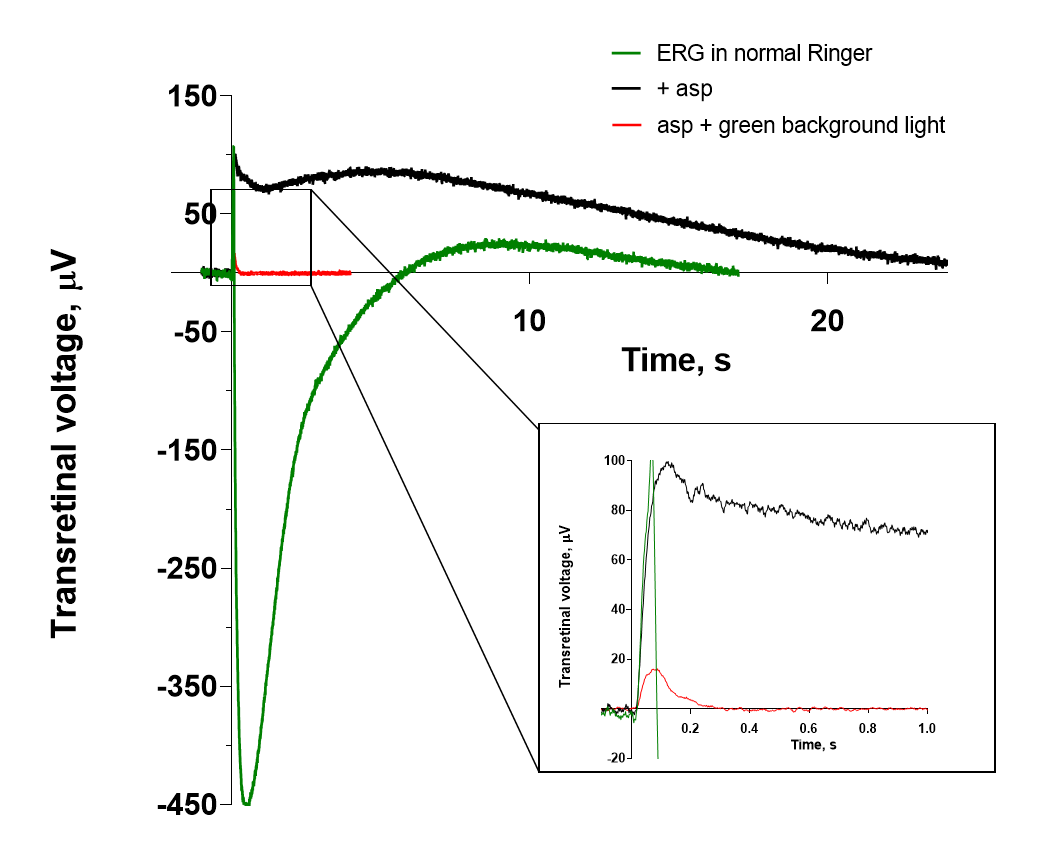


**Figure S4.**  Extraction of the cone component from ex vivo ERG recorded from the frog retina. All three responses were elicited by a brief flash (2 ms, 8•10^4^ photons•μm^-2^). The first response (green curve) was recorded in normal Ringer’s solution and has the typical pattern with a- and b-wave. The second response was recorded after perfusion with normal Ringer’s solution + 10 mM sodium aspartate (black line) and contains only the photoreceptor component. The third response was recorded after turning on the continuous green light (43400 photons•μm^-2^•s^-1^) and it is deemed to contain only the cone response (red line).
